# ESTIMATING THE ECONOMIC IMPACTS OF CLIMATE CHANGE ON 16 MAJOR US FISHERIES

**DOI:** 10.1142/s2010007821500020

**Published:** 2020-12-30

**Authors:** CHRIS MOORE, JAMES W. MORLEY, BRIAN MORRISON, MICHAEL KOLIAN, ERIC HORSCH, THOMAS FRÖLICHER, MALIN L. PINSKY, ROGER GRIFFIS

**Affiliations:** National Center for Environmental Economics, United States Environmental Protection Agency 1200 Pennsylvania Avenue NW (MC 1809T), Washington, DC 20460, USA; Department of Biology, Coastal Studies Institute, East Carolina University, ECU Outer Banks Campus 850 NC 345, Wanchese, NC 27981, USA; Industrial Economics, Incorporated, 2067 Massachusetts Avenue, Cambridge, MA 02140, USA; Office of Atmospheric Programs, United States Environmental Protection Agency, 1200 Pennsylvania Avenue NW (MC 6207A), Washington, DC 20460, USA; Industrial Economics, Incorporated, 2067 Massachusetts Avenue, Cambridge, MA 02140, USA; Climate and Environmental Physics Division (CEP), Physics Institute, University of Bern Sidlerstrasse 5, 3012 Bern, Switzerland; Oeschger Centre for Climate Change Research, University of Bern Hochschulstrasse 4, 3012 Bern, Switzerland; Department of Ecology Evolution and Natural Resources, School of Environmental and Biological Sciences, Rutgers University, New Brunswick, NJ 08901, USA; Office of Science and Technology National Oceanic and Atmospheric Administration (NOAA), 1335 East-West Highway, Silver Spring, MD 20910, USA

**Keywords:** Commercial fisheries, marine species distribution, welfare impacts, climate change, warming ocean temperatures

## Abstract

Observational evidence shows marine species are shifting their geographic distribution in response to warming ocean temperatures. These shifts have implications for the US fisheries and seafood consumers. The analysis presented here employs a two-stage inverse demand model to estimate the consumer welfare impacts of projected increases or decreases in commercial landings for 16 US fisheries from 2021 to 2100, based on the predicted changes in thermally available habitat. The fisheries analyzed together account for 56% of the current US commercial fishing revenues. The analysis compares welfare impacts under two climate scenarios: a high emissions case that assumes limited efforts to reduce atmospheric greenhouse gas and a low emissions case that assumes more stringent mitigation. The present value of consumer surplus impacts when discounted at 3% is a net loss of $2.1 billion (2018 US$) in the low emissions case and $4.2 billion in the high emissions scenario. Projected annual losses reach $278–901 million by 2100.

## 1. Introduction

Climate change has resulted in significant impacts on biological communities in marine ecosystems. These changes have included restructuring of species composition (Fodrie *et al.*, 2010; Wernberg *et al.*, 2016; Flanagan *et al.*, 2019), unprecedented changes in species phenology (Edwards and Richardson, 2004; Mills *et al.*, 2013; Staudinger *et al.*, 2019), and geographic shifts in species distributions (Pinsky *et al.*, 2013; Poloczanska *et al.*, 2013). Evidence for the redistribution of marine species has also been observed in global fisheries, with changes in catch composition consistent with poleward shifts in species distributions (Cheung *et al.*, 2013). Further, during the last century climate change, along with other stressors, has reduced potential fisheries yields at a global scale (Free *et al.*, 2019), suggesting widespread and negative economic implications. Regional fisheries impacts have also occurred, including declines in accessibility of target species to fisherman (Young *et al.*, 2018; Pinsky and Fogarty, 2012; Hughes *et al.*, 2015), changes in stock productivity (Hare and Able, 2007; Bell *et al.*, 2014; Pershing *et al.*, 2015), and regional conflicts over quota allocations as the species shift across jurisdictional lines (Dubik *et al.*, 2019; Spijkers and Boonstra, 2017).

Future projections of climate change impacts in the coming century suggest a global loss of biomass in the oceans, especially at the middle and lower latitudes (Lotze *et al.*, 2019). Potential landings are also projected to decline during the 21st century on many of the most valuable fishing grounds (Cheung *et al.*, 2010). Further, factors other than changes in landings, such as changes in catch composition toward lower-value species, might lead to dramatic losses of fisheries revenues (Lam *et al.*, 2016). Major changes in the geographic distribution of marine species are also projected during the coming century, as a result of shifts in preferred temperatures (Cheung *et al.*, 2009; Wisz *et al.*, 2015; Morley *et al.*, 2018). Such shifts in distribution will challenge fisheries management as the species move across jurisdictional boundaries (Haynie and Pfeiffer, 2012; Pinsky *et al.*, 2018).

While global-scale economic analyses of the potential impacts of climate change on fisheries are important (Lam *et al.*, 2016), the regional-scale assessments are critical to inform policymakers of the expected impacts (e.g., Jones *et al.*, 2015). Indeed, in the US, projections of the economic impacts of climate change on fisheries have been identified as a federal research priority (Busch *et al.*, 2016). The US is one of the highest producers of wild-caught marine seafood globally (FAO, 2018), and some of the most rapidly increasing ocean temperatures in the world are off the US coast (Burrows *et al.*, 2011). Studies of the Gulf of Maine have documented significant increases in ocean temperatures and evidence of marine heat waves (Mills *et al.*, 2013; Pershing *et al.*, 2015), which satellite observations suggest are becoming more frequent, intense, and extensive (IPCC, 2019). Future projections of thermal habitat for hundreds of species on the North American continental shelf predict major shifts in distribution for many economically important species (Morley *et al.*, 2018). These high-resolution projections (0.05° latitude and longitude) can be used to estimate the economic impacts on US marine fisheries, mirroring an approach that has been used at a global scale (Lam *et al.*, 2016).

This study follows the analytic framework established by the Climate Change Impacts and Risk Analysis (CIRA) project for quantifying and monetizing potential climate change impacts across various sectors in the US. The CIRA project examines both the potential effects of climate change on the US and the potential economic impacts of mitigating global greenhouse gas (GHG) emissions. CIRA analyses published to date have included only limited consideration of the projected effects of climate change on the commercial fishing and ecosystem services (EPA, 2015, 2017). Here, we leverage an ongoing research (i.e., Morley *et al.*, 2018) to address this gap and broaden the understanding of approaches to estimating the potential economic effects of climate change on the commercial fishing sector. The analysis first characterizes the potential economic impact of projected changes in the annual landings of 177 commercially harvested marine species from 2021 to 2100, based on the use of five general circulation models (GCMs) to project changes in each target species’ thermally available habitat within the US Exclusive Economic Zone (EEZ). It then focuses on 16 US fisheries that together account for 56% of the current US commercial fishing revenues. Consistent with the recently completed Fourth National Climate Assessment of the U.S. Global Change Research Program, the analysis compares welfare impacts for these fisheries under two atmospheric GHG concentration scenarios: Representative Concentration Pathway (RCP) 8.5, a higher emissions case that assumes limited efforts to reduce atmospheric GHG; and RCP 4.5, a lower end case that assumes more stringent mitigation (USGCRP, 2018).

## 2. Methods and Data Sources

### 2.1. Projected changes in thermal habitat

#### 2.1.1. Approach

The analysis of projected changes in thermal habitat is based on the methods described by Morley *et al.* (2018) to examine the potential impacts of ocean warming on the geographic distribution of 686 marine species on the North American continental shelf from 2021 to 2100. This study examined future habitat shifts across a suite of 16 GCMs within the RCP 2.6 and 8.5 scenarios. To be consistent with the framework of the multi-sectoral CIRA 2.0 project (EPA, 2017), this analysis uses five GCMs. The models and their developers include:

- CanESM2, Canadian Centre for Climate Modelling and Analysis;
- CCSM4, National Center for Atmospheric Research;
- GISS-E2-R, NASA Goddard Institute for Space Studies;
- HadGEM2-ES, Met Office Hadley Centre;
- MIROC5, Atmosphere and Ocean Research Institute, National Institute for Environmental Studies, and Japan Agency for Marine-Earth Science and Technology.

A variety of factors were considered in selecting these five models for impacts analysis in the US, including their structural independence, quality, and ability to reasonably capture variability in temperature and precipitation outcomes (EPA, 2017). The first three GCMs listed were among the 16 employed in the original analysis of the impacts of ocean warming conducted by Morley *et al.* (2018); HadGEM2-ES and MIROC5 were added to ensure consistency with CIRA’s modeling framework. These five GCMs were run within two carbon emissions scenarios: RCP 8.5 assumes limited efforts to reduce greenhouse gas emissions and results in more ocean warming, while RCP 4.5 assumes more stringent GHG mitigation and less warming.

Projected changes in annual species distribution during the 21st century under the 10 potential future climates (two RCPs × five GCMs) were based on statistical thermal niche models for each species (Morley *et al.*, 2018). The niche models were based on 20 long-term bottom trawl surveys, which recorded data on the species’ presence or absence, as well as biomass (*N* = 136,044 samples). These surveys are conducted annually by the United States and Canada and encompass most of the continental shelf of these two countries. The niche model predictor variables included mean seasonal sea surface temperature (SST) and sea bottom temperature (SBT); annual maximum SST and SBT; annual minimum SBT; seafloor rugosity (i.e., local variation in depth); and sediment grain size. Environmental data came from multiple sources and were associated with survey catch data based on the date and location of each sample (Morley *et al.*, 2018).

Generalized additive models (GAMs) were used to quantify each species’ thermal niche. GAMs provide an effective way to quantify species’ relationships with environmental variables because they allow complex nonlinear associations and require no *a priori* assumption about the shape of these relationships (Brodie *et al.*, 2020). Previously, two GAMs were fitted for each species, one that modeled the probability of occurrence using presence and absence data and the one that modeled log-biomass using only samples where a species was present; the product of these two GAMs was used for predictions (i.e., the delta-biomass approach). A more recent analysis on the influence of the niche modeling approach on species habitat projections showed that probability of occurrence approaches, as compared to methods that predict biomass, more often had better predictive performance when tested with independent historic data (Morley *et al.*, 2020). Therefore, for this analysis we based our projections of change in habitat distribution on the modeled probability of occurrence, not biomass. For each species, model skill of the GAMs was tested with independent trawl survey data using the area under the receiver operator curve (AUC) statistic, which compares predicted versus observed species occurrence. Only species with AUC scores greater than 0.75 were retained for analysis, which is a limit-value that has been shown to indicate models that are effective at modeling the species distribution (Elith *et al.*, 2006).

**Figure 1.**
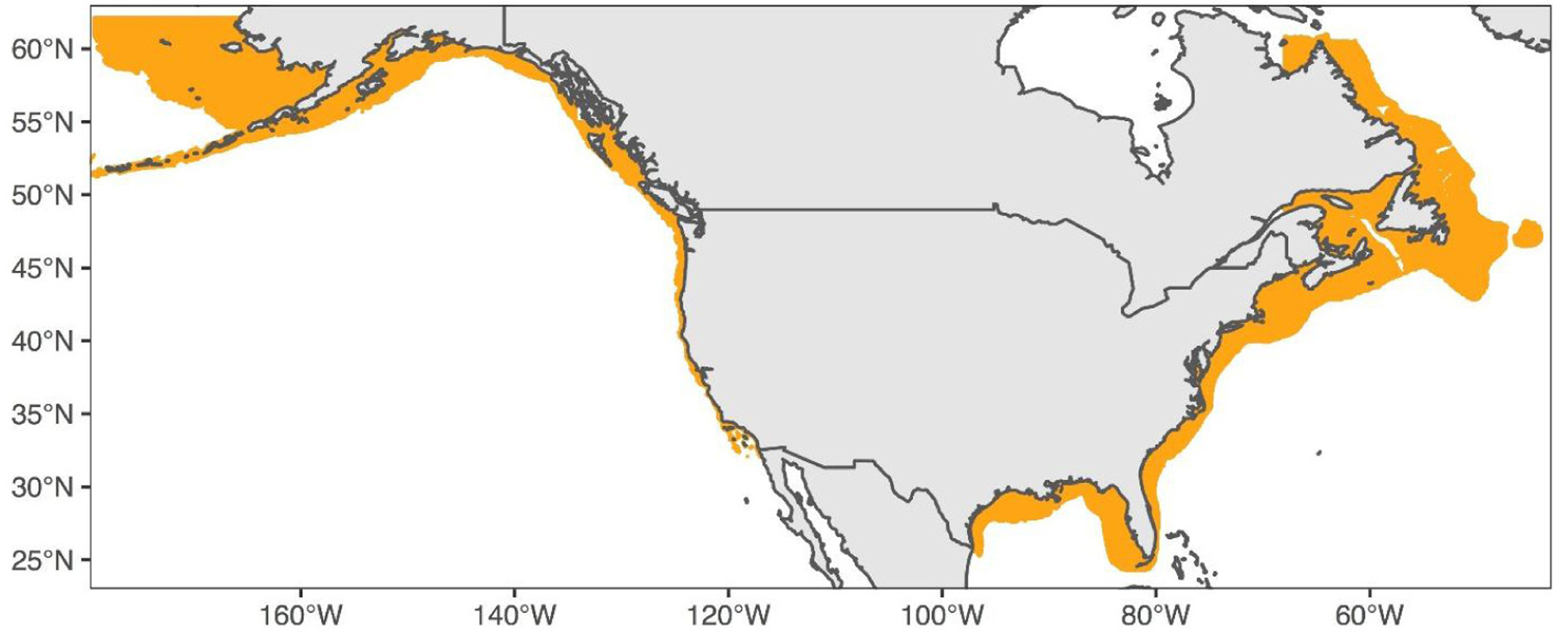
Projection grid for thermal habitat analysis.

Future projections of species distributions were based on the annual forecasts for mean summer (July–September) ocean conditions and represent an expanded version of the dataset used in Morley *et al.* (2018). Climate projections that were added for this study (i.e., RCP 4.5 and two new GCMs) were processed in an identical manner to Morley *et al.* (2018). Specifically, projected changes in ocean temperatures from GCMs were downscaled to a ~0.25° latitude and longitude grid based on a mean temperature climatology that was developed from the SODA3.3.1 ocean reanalysis product for 1995–2014 (Carton *et al.*, 2016). The modeled historic climate data that was used for downscaling temperature projections was highly correlated to *in-situ* historic observations of sea surface [slope (se) = 0:91 (0.001), *p* < 0:001, DF = 102,048, *r*^2^ = 0:90] bottom [slope (se) = 1 (0.001), *p* < 0:001, DF = 120,859, *r*^2^ = 0:86] temperatures. The climate projection grid was further refined to 0.05° latitude and longitude based on the spatial resolution of the seafloor data; depth was limited to 400 m or shallower. The resulting projection grid consisted of 65,826 individual cells on the Pacific coast, 69,209 on the Atlantic coast, and 13,383 in the Gulf of Mexico (Fig. 1). This projection grid was then restricted to waters within the US EEZ, where the US has sovereign fishing rights, and partitioned into four regions for analysis: US East Coast, Gulf of Mexico, US West Coast, and Alaska.

For each species, a set of 10 (two RCPs × five GCMs) annual-summer thermal habitat distributions from 2007 to 2100 were developed. Annual grid cell values were aggregated by averaging the projections within five multi-year bins, which included a baseline period of 2007–2020 and four future time periods: (T1) 2021–2040, (T2) 2041–2060, (T3) 2061–2080, and (T4) 2081–2100. During each time period, total available thermal habitat within the US regions was calculated as the sum of all grid cell values (i.e., modeled probability of occurrence). The percentage change in future thermal habitat availability was calculated based on the differences between the baseline and future time periods. For each future time period, we then calculated an ensemble mean value across GCMs for RCPs 4.5 and 8.5. This process produced a total of 1085 unique species-region projections for initial consideration in our economic analysis.

#### 2.1.2. Limitations of species distribution projections

The projected changes in species distribution suggested by this modeling exercise reflect only the predicted changes in the areal extent and quality of potentially suitable habitat. The analysis does not employ predictions of changes in the absolute biomass of any stock and excludes many factors that may influence species abundance, such as potential changes in primary productivity, species interactions, population dynamics, or fisheries management. The boundaries of the projection grid are also a limiting factor, particularly in Northern Alaska, and may affect the results for the species found primarily in that region. In the Gulf of Mexico, the thermal niche models may not adequately characterize the upper temperature limits for some species, which may be reached at temperatures above the maximums observed in the underlying trawl surveys. In addition, the analysis does not account for a variety of other factors that may influence marine habitat or species productivity. These include but are not limited to potential changes in weather or ocean circulation patterns, changes in sea level, changes in nutrient loads, or changes in ocean acidity. Models that consider such factors are under development but have yet to be applied at a broad scale.

Despite these limitations, the modeling exercise provides useful insights to potential changes in suitable habitat for hundreds of species across a geographic range that includes much of the US EEZ and many of the nation’s most highly valued fisheries. The breadth of the analysis, coupled with the information it provides on potential changes in the habitat, offers a useful basis for a first-order analysis of the effects of increased sea temperatures on the commercial harvests of economically important species.

### 2.2. Economic screening analysis

#### 2.2.1. Overview of data

As a first step in assessing the potential economic impacts of changes in species distribution on the commercial fishing sector, we obtained data from the National Marine Fisheries Service (NMFS) on commercial landings in the United States from 2007 to 2016, disaggregated by species and region (East Coast, Gulf Coast, West Coast, and Alaska). The NMFS dataset reports both the quantity (pounds) and dollar value (i.e., ex-vessel revenue) of landings in these regions, which together accounted for 97.4% of the value of US commercial landings in 2016 (NMFS, 2017). Hawaii, the Great Lakes region, and the US territories account for the balance of US landings.

For each species-region, we calculated the mean annual landings by weight and value for 2007–2016, using the Consumer Price Index (CPI) to convert the annual data on ex-vessel revenues to 2018 US dollars (BLS, 2018). After collapsing the data to a single record for each species-region, the resulting dataset consisted of 883 records. Table 1 summarizes the data on landings by region.

We employed an automated process to match the 883 species-region records on commercial landings to the 1085 species-region records for habitat projections, using taxonomic nomenclature. This resulted in a match for 247 records. We then initiated a manual review of the remaining records to identify potential matches the automated process might have missed.

We were unable to link 88 records from the NMFS dataset to a habitat projection because the records represent commercial landings for more than one species (e.g., skates). NMFS frequently reports landings at a higher taxonomic level, in some cases because the taxonomic identification in port is difficult — particularly when the species that are physically similar are landed together — and in others to protect the confidentiality of industry participants (i.e., when only one or two vessels account for all landings of an individual species). The aggregated data provided by NMFS cannot be disaggregated by species. This narrowed the scope of our analysis to individual species for which comparable commercial landings data are available.

We sorted the remaining 548 unmatched species-region records from the NMFS dataset by economic value. We set aside 414 of these records — all those with an average annual value of less than $100,000, which together represent approximately 0.1% of total revenues — as being of minimal economic significance. We reviewed the remaining 134 records to attempt to match them to the available future habitat projections. This manual review identified five cases in which the use of taxonomic synonyms by the two datasets had prevented an automated match. We confirmed that projections of changes in thermally available habitat were not available for the species represented by the remaining 129 records. These included some commercially important species that are not effectively sampled in the biological surveys upon which the niche modeling is based (e.g., eastern oyster, Atlantic surf clam, Caribbean spiny lobster, and multiple species of salmon and tuna).

Table 2 shows the disposition of the 883 NMFS landings records from the matching process. The results are shown for both the count of records and with respect to average annual ex-vessel value. As the exhibit indicates, projections of changes in thermally available habitat are available for 252 species-region records. These records represent a total of 177 species and account for 70.8% of the average annual commercial fishing revenues (2007–2016) in the four regions analyzed. Our screening assessment of the potential impact of ocean warming on commercial landings focuses on these species.

The screening analysis provides good coverage of high-value fisheries. As Table 3 shows, the species for which projections of changes in habitat are available include nine of the nation’s 10 leading fisheries from 2007 to 2016, as measured by the average annual revenue: American lobster (*Homarus americanus*); sea scallops (*Placopecten magellanicus*); walleye pollock (*Theragra chalcogramma*); white shrimp (*Litopenaeus setiferus*); Pacific cod (*Gadus macrocephalus*); brown shrimp (*Farfantepenaeus aztecus*); Dungeness crab (*Metacarcinus magister*); Pacific halibut (*Hippoglossus stenolepis*); and blue crab (*Callinectes sapidus*). The exception is sockeye salmon (*Oncorhynchus nerka*), which ranked fourth in the average annual revenue over the period of interest.

Table 4 provides an overview of the availability of data for the screening analysis by region. As the table indicates, the species for which habitat projections are available account for nearly 80% of the average annual ex-vessel revenues on the East Coast. Coverage is somewhat lower in the other three regions, where the species for which habitat projections are available account for between 63% and 68% of the average annual revenue.

#### 2.2.2. Projections of potential changes in landings

**Figure 2.**
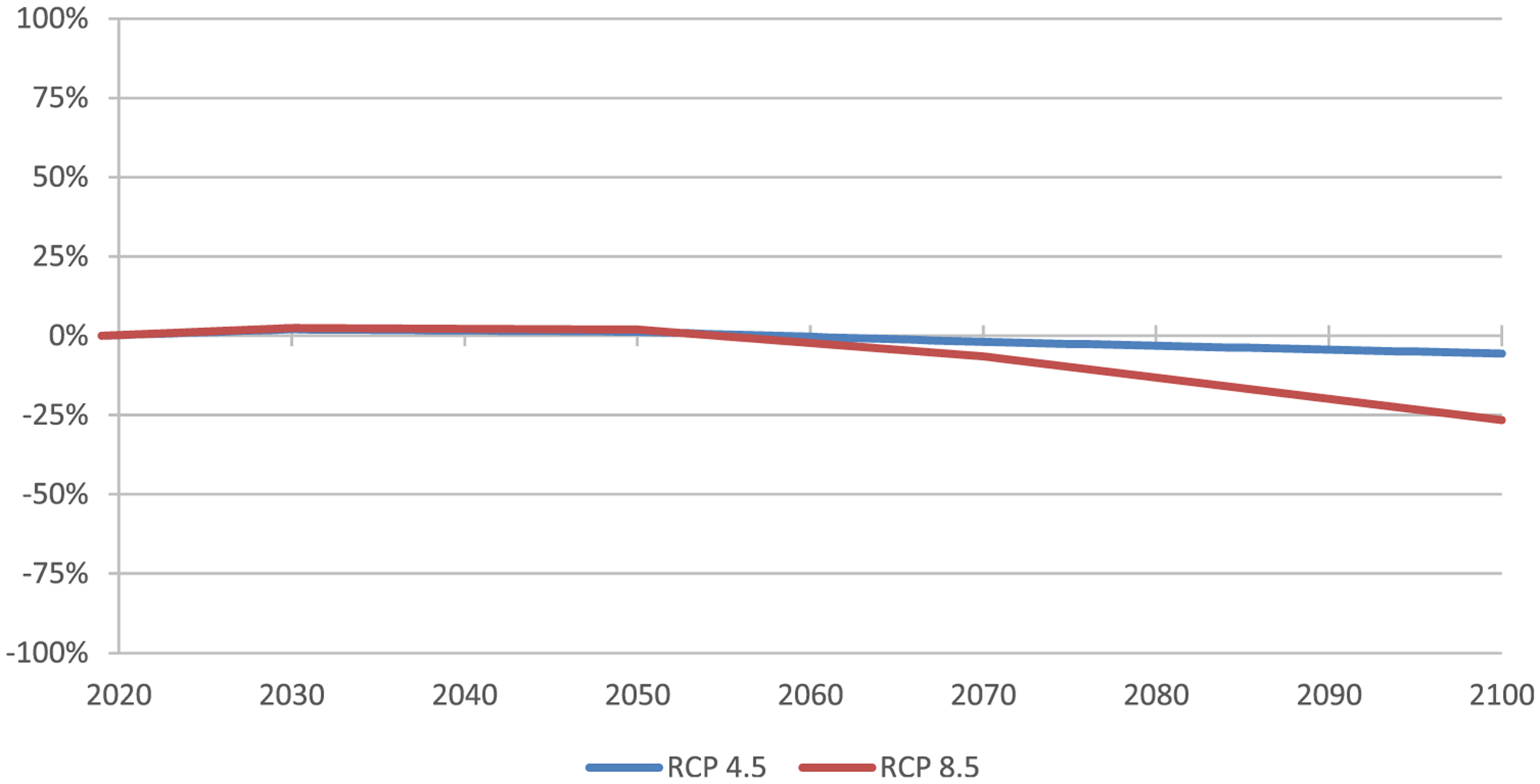
Projected changes in thermally available habitat, American lobster, East Coast: Five-GCM mean.

**Figure 3.**
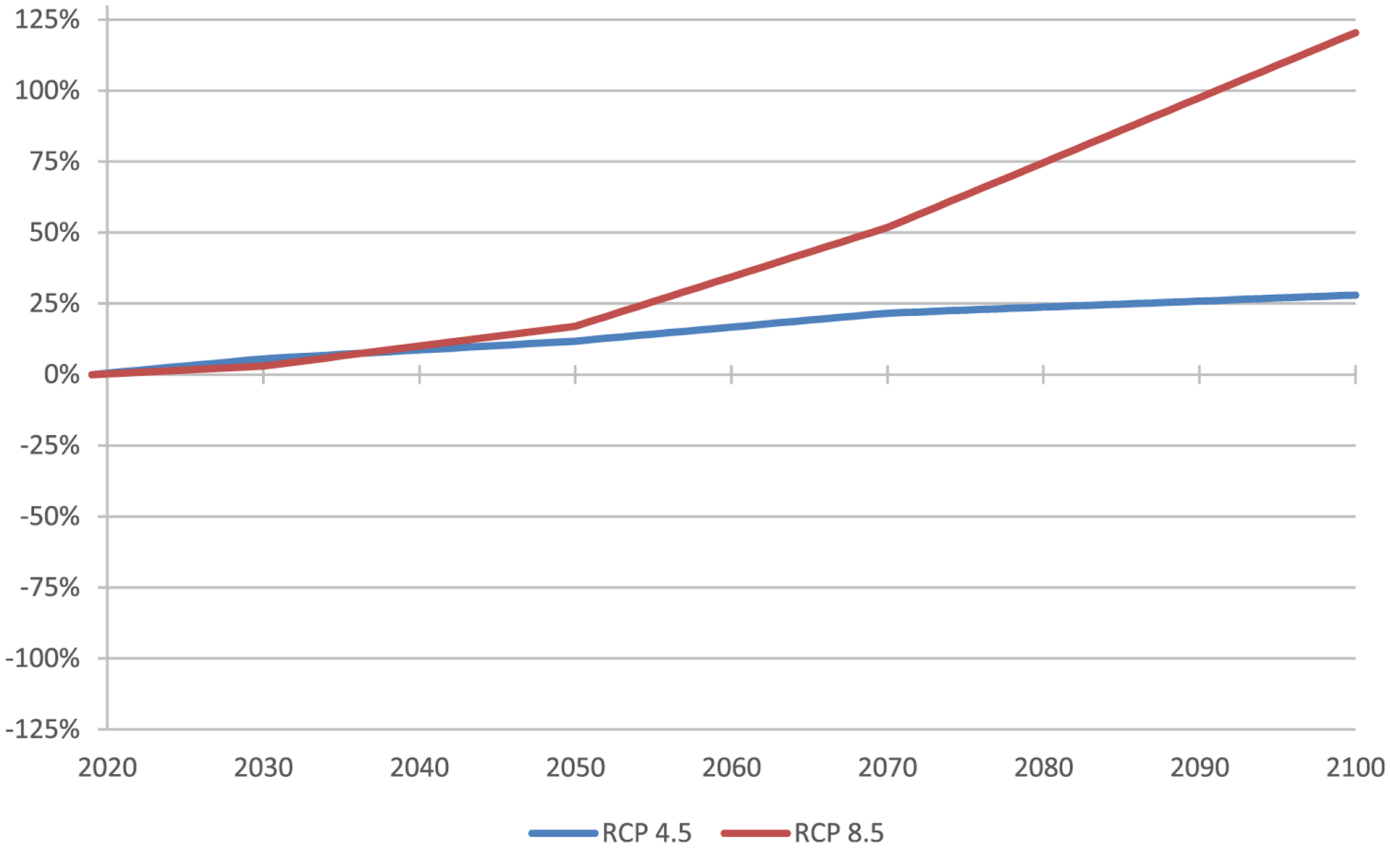
Projected changes in thermally available habitat, blue crab, Gulf of Mexico: Five-GCM mean.

To conduct the screening assessment, we focused on the 252 species-region records from the NMFS dataset for which the projected changes in thermally available habitat are available. Figure 2 provides an example of these projections, showing the predicted changes in thermally available habitat for American lobster within the Atlantic region of the US EEZ, as represented by the five-GCM mean. The figure illustrates the predicted changes in habitat for both RCP 4.5 and RCP 8.5 from 2021 to 2100. The projections show relatively little net change under RCP 4.5 through the end of the century, but a decline under RCP 8.5 beginning mid-century. Figure 3 provides a second example, illustrating the projected changes in available habitat for blue crab in the Gulf of Mexico. In this case, the available habitat is projected to increase under both RCP 4.5 and RCP 8.5 from 2021 through the end of the century. Under the latter scenario, the thermally available habitat for blue crab is projected to more than double.

The implication of changes in thermally available habitat for commercial fishing landings is difficult to predict. The availability of suitable habitat clearly influences species abundance, but the abundance of any species is also a function of primary productivity, interactions with other species, population dynamics, fisheries management measures, and other factors that are difficult to model at a broad geographic scale. Similarly, commercial landings are dependent not only on species abundance but also the intensity of fishing effort, which is in turn a function of market forces, changes in technology, and fisheries management regimes at the state and national levels. Our analysis does not attempt to predict the complex interactions among these variables over the course of the next 80 years. Instead, it considers the potential economic implications of predicted changes in sea temperature assuming a direct relationship over time between changes in the thermally available habitat of a species and commercial landings of that species. The analysis serves as an exploratory assessment rather than a predictive one, for assessing the direction and approximate magnitude of potential changes in landings given the anticipated climate-related changes in thermally available habitat. Its findings should be interpreted and applied with this intent in mind.

As the initial step in the screening analysis, we apply our projection of the percentage change in thermally available habitat for each species in each region, as represented by the mean change in thermally available habitat predicted by the five GCMs, to our baseline estimate of annual landings, as represented by the 2007–2016 mean (2018 US$). This generates a time series of annual landings in each region from 2021 to 2100 for each species analyzed. At this stage of the analysis we ignore the potential effect of changes in supply or changes in real income on the ex-vessel prices. Our objective is to develop a first-order estimate of potential economic impacts and to identify an analytically tractable subset of species that drive the projected results. This subset will become the focus of a more rigorous analysis of potential impacts, which accounts for the effect of changes in supply and income on market prices.

To provide a general assessment of the direction and potential magnitude of impacts in each region, we compare the discounted present values of projected landings under RCP 4.5 and RCP 8.5 over the period of interest (2021–2100) to the discounted present value of landings if maintained at the recent historical levels (i.e., the average annual ex-vessel value from 2007 through 2016). Consistent with other CIRA analyses, the present value calculation employs a real annual discount rate of three percent (EPA, 2017). Selection of this rate is supported by the literature on valuing changes in private consumption and the treatment of intergenerational equity when discounting impacts over long time horizons (OMB, 2003; Scarborough, 2011).

Table 5 presents the projected changes in present value of ex-vessel revenues under RCP 4.5, holding the ex-vessel prices constant and assuming that the landings of each species analyzed change in direct proportion to the projected changes in thermally available habitat. The analysis indicates a loss of approximately $1 billion, a 0.9% decline in the present value of landings relative to the baseline. As the table shows, the projected impacts differ by region. On a present value basis, changes in thermally available habitat off the East Coast and Gulf Coast are projected to have a positive impact on commercial landings. In contrast, changes in thermally available habitat in the Pacific waters of the US EEZ are projected to have a negative impact, both in the Alaska region and off the West Coast.

Table 6 shows a similar set of estimates for RCP 8.5. In this case, the projected decline in the present value of ex-vessel revenues is $1.6 billion, a 1.4% loss relative to the baseline. The impact in Pacific waters remains negative, particularly in the West Coast region, but the projected impact on landings elsewhere is mixed. The analysis shows a decidedly positive impact in the Gulf region, largely due to projected increases in the available habitat for blue crab and white shrimp; we note that these projections may be overstated, because the thermal niche models for these species may not effectively capture their upper temperature limits. In contrast, the impact on the East Coast is slightly negative, due primarily to projected reductions in the available habitat for high-value species like sea scallops and American lobster.

Tables 7 and 8 illustrate the results of the screening analysis for the 20 highest-value fisheries for which the habitat projections are available. Table 7 shows that under the RCP 4.5 scenario, the present value of ex-vessel revenues is projected to increase for 11 of the 20 fisheries and to decrease for the others. The greatest absolute impact is projected for the snow crab fishery, where the present value of landings from 2021 to 2100 is projected to decline by more than $1 billion (30.6%) compared to the present value of landings if maintained at the recent historical levels (i.e., the average annual ex-vessel value from 2007 through 2016). In contrast, the analysis suggests that the present value of landings of white shrimp could increase by more than $670 million (9%).

Table 8 provides a comparable set of estimates for RCP 8.5. For 17 of the 20 fisheries, the direction of the projected impact on the present value of landings remains the same. Moreover, as might be anticipated for most of these fisheries, the magnitude of projected impacts under RCP 8.5 is greater than under RCP 4.5. For three fisheries, however, the direction of the projected impact changes. In the case of the American lobster and sea scallop fisheries, the projected impact switches from positive to negative, as small gains in habitat in the East Coast region early in the century are offset by greater losses in habitat toward the century’s end. In contrast, the projected impact for Chinook salmon landings switches from negative to positive. In this case, more rapid warming in the RCP 8.5 scenario leads to an earlier and more substantial increase in thermally available ocean habitat in the Alaska region, offsetting a loss of ocean habitat along the West Coast. Note that the analysis does not consider the availability or condition of the freshwater habitat on which the anadromous species like Chinook salmon depend, an important consideration in projecting changes in the future landings of such species.

#### 2.2.3. Implications for economic welfare analysis

**Figure 4.**
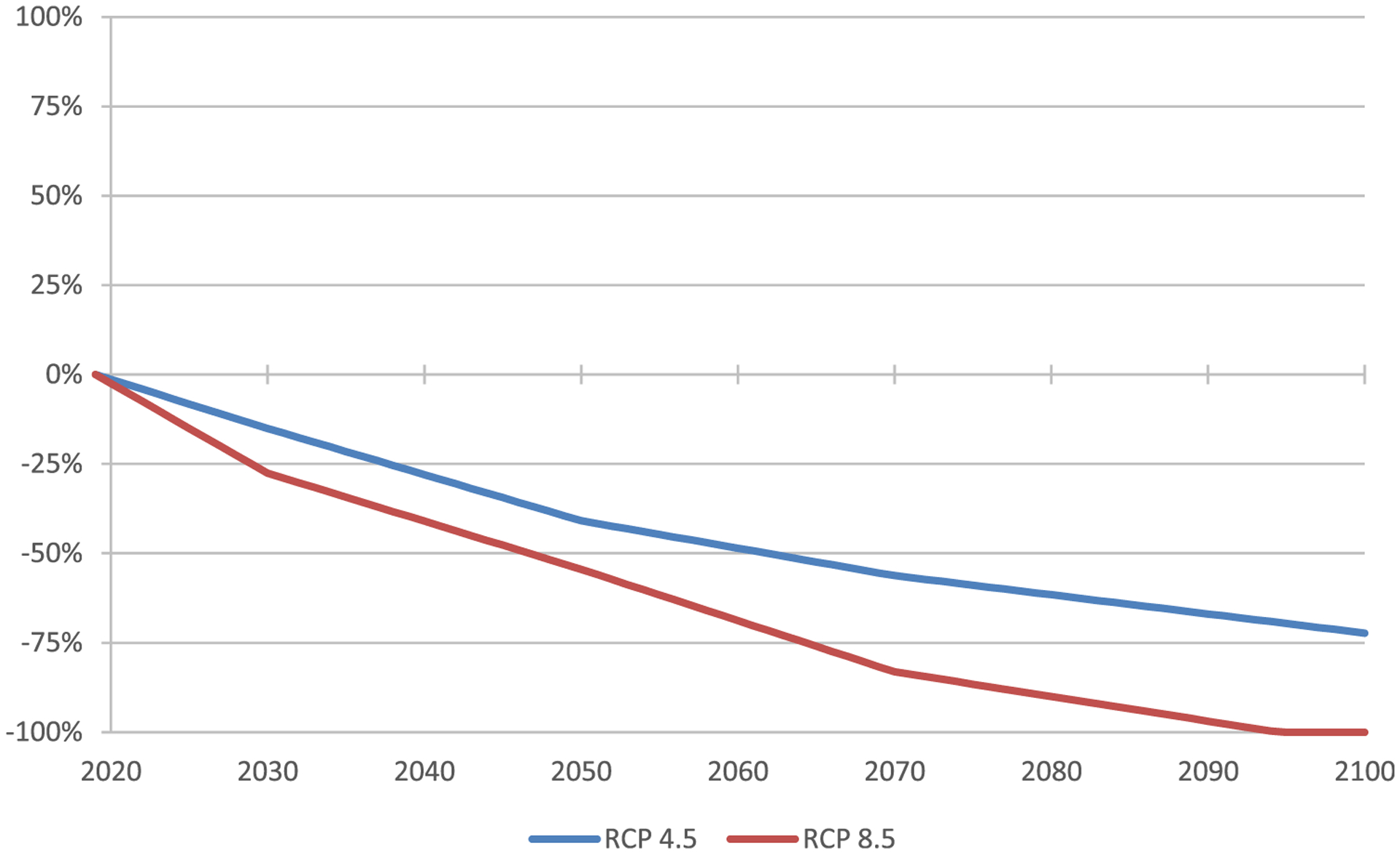
Projected changes in thermally available habitat, snow crab, Alaska: Five-GCM mean.

In addition to providing general insight to the potential effects of climate change on the future landings of commercially harvested species, the screening analysis helped to guide the selection of fisheries considered in our assessment of consumer welfare impacts. To ensure that the welfare assessment would be analytically tractable, we limited its scope to 16 species that could be equally divided into four categories, each of which would contain commodities that the consumers might consider close substitutes. Given the limited number of species the analysis could consider, we also chose to focus, to the extent possible, on fisheries that account for the greatest share of current ex-vessel landings. One exception to this selection process was snow crab, a species for which our analysis of RCP 8.5 projects a complete loss of thermally available habitat within the areas modeled by the end of the century (see Fig. 4). The implication of this finding — that landings of snow crab would fall to zero by the end of the century — is analytically intractable. More importantly, the thermally available habitat for snow crab in the Bering Sea shows a strong potential to shift northward, beyond the northern boundary of our projection grid. This raises concern that the geographic limits of the habitat analysis may lead us to overstate the impact of rising temperatures on future landings. These factors led us to exclude snow crab from the welfare analysis, resulting in selection of the following species, by fishery category:

- Lobster/crab: American lobster, blue crab, Dungeness crab, and Florida stone crab (claws);
- Shrimp/mollusk: sea scallop, white shrimp, brown shrimp, and California market squid;
- High-value fish (mean ex-vessel price greater than $0.75 per lb, 2018 US$): Pacific halibut, sablefish, Chinook salmon, and summer flounder;
- Low-value fish (mean ex-vessel price less than $0.75 per lb, 2018 US$): walleye pollock, Pacific cod, yellowfin sole, and chum salmon.

Table 9 lists these species, noting the baseline rank of each fishery by ex-vessel value. As it indicates, the scope of the analysis includes 16 of the 20 fisheries in the screening analysis dataset with the greatest average annual ex-vessel revenues. In aggregate, the revenue associated with these 16 fisheries accounts for 82% of the dataset’s baseline total and 56% of commercial landings in the four regions analyzed. Moreover, the 16 fisheries include those the screening analysis suggests might experience an increase in the present value of landings in response to warming temperatures, as well as those that might experience a decrease. Thus, the welfare analysis not only captures the impacts on fisheries that are currently economically important, but also reflects the expected variation in the implications of rising sea temperatures for different species.

**Figure 5.**
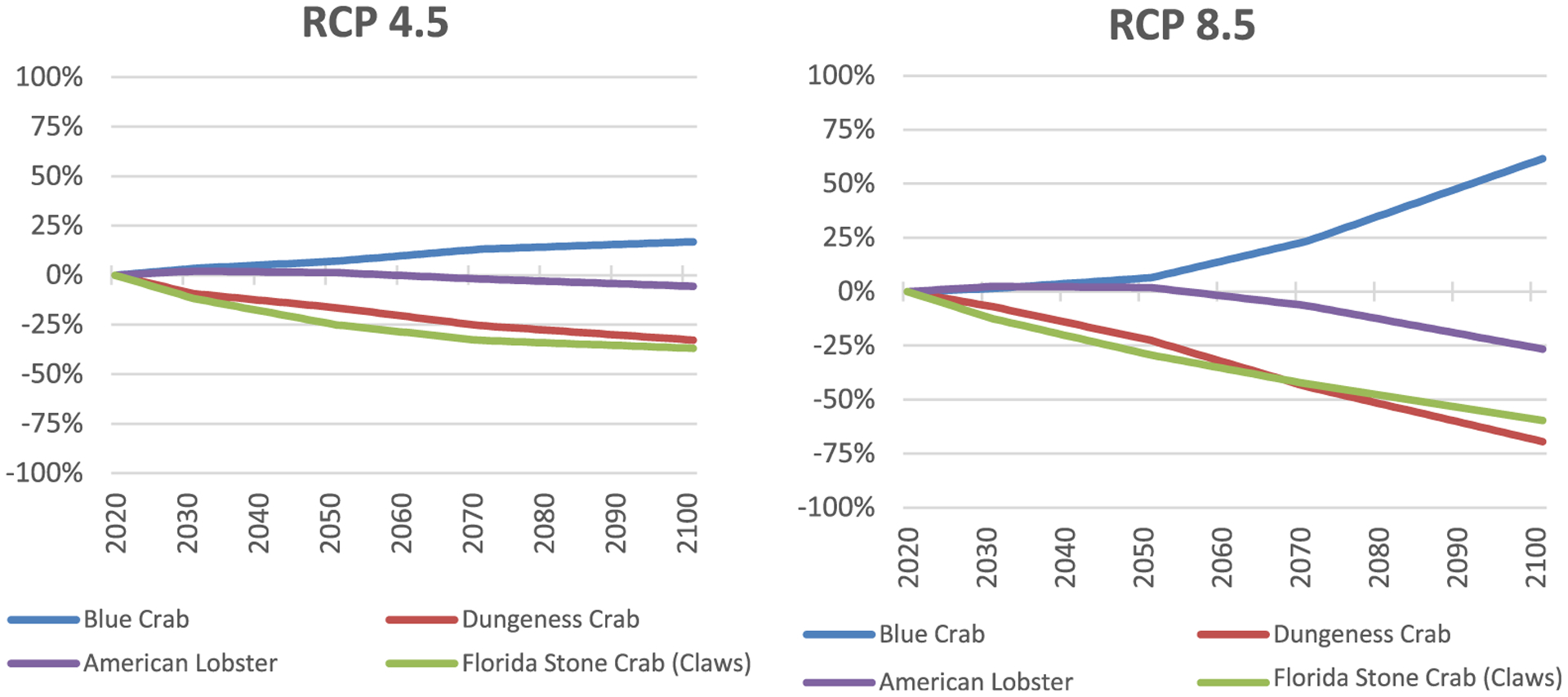
Projected changes in commercial harvests of key lobster/crab species: Five-GCM mean.

**Figure 6.**
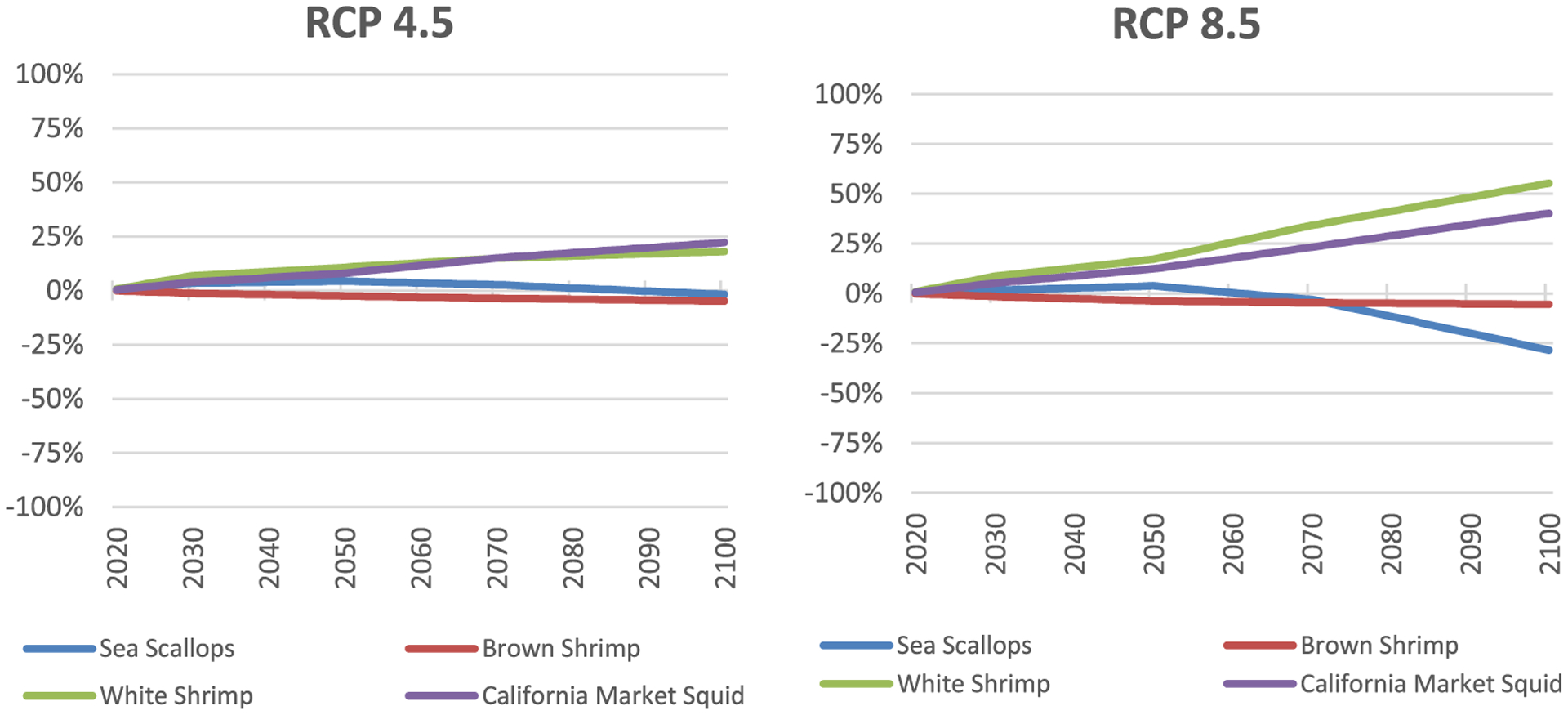
Projected changes in commercial harvests of key shrimp/mollusk species: Five-GCM mean.

**Figure 7.**
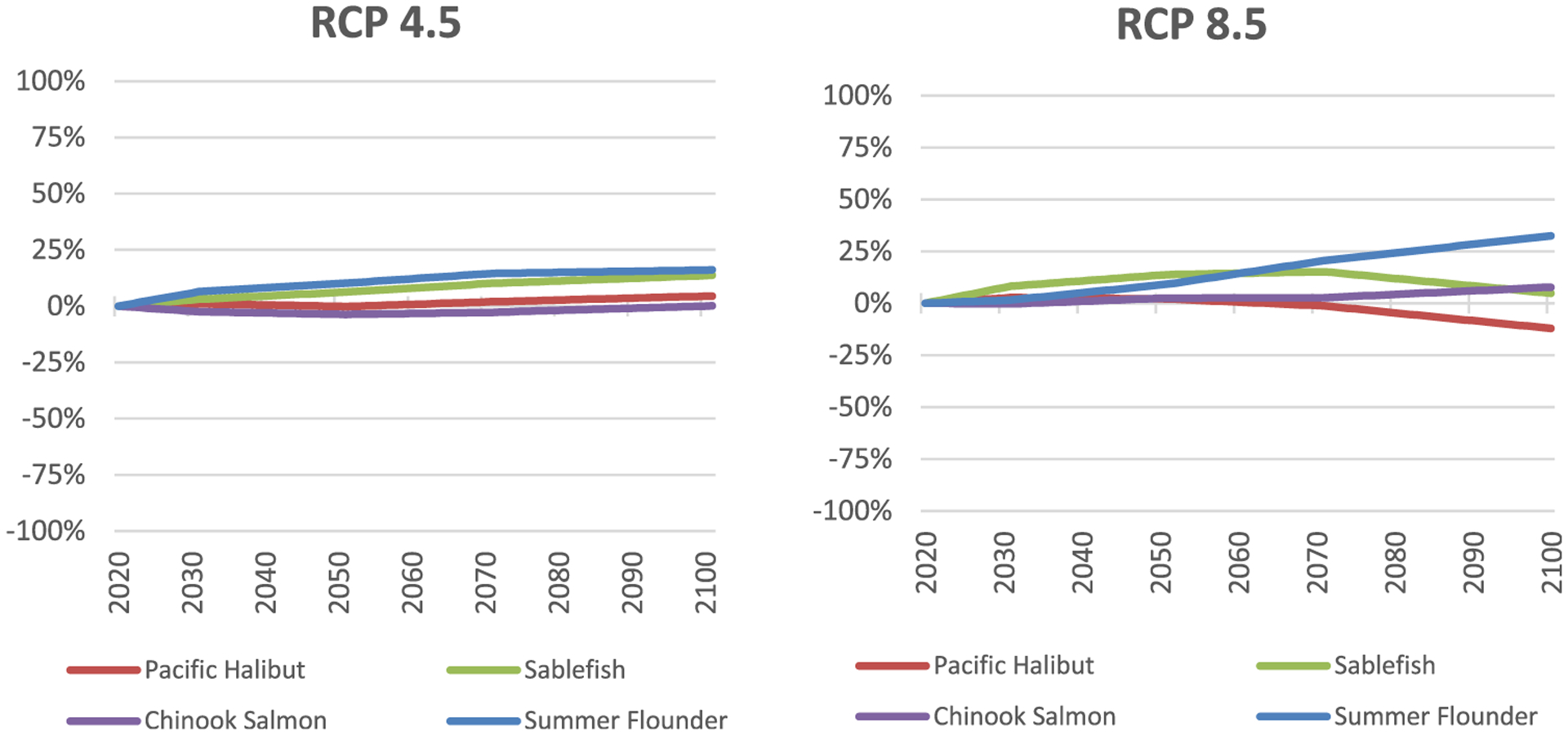
Projected changes in commercial harvests of key high-value fish species: Five-GCM mean.

**Figure 8.**
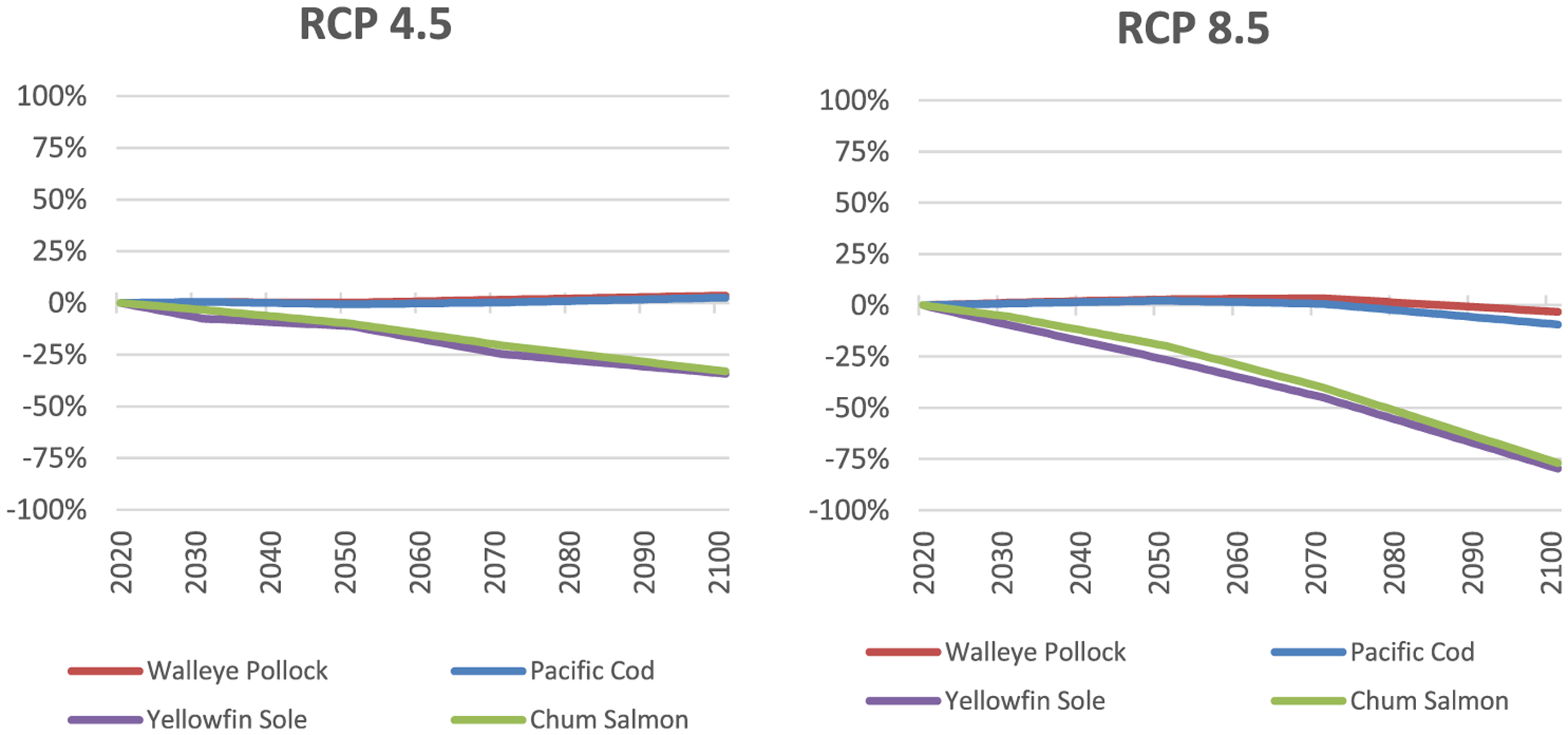
Projected changes in commercial harvests of key low-value fish species: Five-GCM mean.

Figures 5–8 present the mean projected changes in annual harvests (in percentage terms) for the 16 modeled species, with separate figures for each fishery group. There are two panels in each figure, one for RCP 4.5 and another for RCP 8.5. Tables 10 and 11 present the baseline harvests (in millions of lb) for these 16 species and the projected harvests in 2050 and 2090 for the two climate scenarios. The tables also report the 95% confidence intervals around the projected change in harvest, based on a Monte Carlo simulation that considered two factors: (1) variation in annual landings from 2007 through 2016; and (2) variation in the predicted change in thermally available habitat across the five GCMs. As these tables and figures show, the projected changes in landings under RCP 8.5 are generally more rapid and pronounced than those under RCP 4.5, particularly toward the end of the century. In the high-end case, the projected increase or decrease in landings by 2090 for several species approaches or exceeds 50%. Changes of this magnitude suggest substantial shifts in the distribution of seafood products available to consumers. In the discussion that follows, we examine the implications of these changes for consumer welfare.

#### 2.2.4. Limitations of the economic models

As previously noted, this analysis excludes many factors that may influence species abundance and commercial landings, such as potential changes in primary productivity, species interactions, population dynamics, or fisheries management. In addition, because the approach focuses on potential changes in the landings of species that are already commercially harvested, it does not account for the possibility that an increase in the abundance of other species could lead to the development of new fisheries. This type of development would help to offset potential losses in economic welfare attributable to a decline in the productivity of established fisheries.

An additional limitation of the analysis concerns our ability to characterize the uncertainty around the projected changes in landings. The confidence intervals presented in Tables 10 and 11 are based on a Monte Carlo simulation that considered two factors: (1) variation in annual landings from 2007 through 2016; and (2) variation in the predicted change in thermally available habitat across the five GCMs. These confidence intervals do not reflect other sources of uncertainty in the GCMs’ projections of changes in the thermally available habitat, nor do they account for the impact of the considerations noted in the previous paragraph.

Finally, it is important to note that while we are examining consumer surplus, the quantity and value data we use to estimate our model are taken from domestic dockside transactions. The supply chain from the fishing vessel to the consumer’s table is complex. The US imports the majority of its seafood and an increasing fraction is produced in aquaculture. Given some imported seafood and aquaculture are close substitutes for domestic wild harvest, omitting them from our analysis could affect our elasticity and welfare estimates. To the extent that the data we use in our analysis is generated in markets that include imports and aquaculture, our demand responses to changes in domestic wild harvest are consistent so long as we assume those other supplies are held constant. The effects of climate change on the imported seafood and the emergence of aquaculture are not addressed in this paper; we simply recognize them as limitations to a more holistic analysis.

## 3. Analysis of Welfare Impacts

### 3.1. Modeling approach

Given the expected changes in annual harvests for the 16 modeled species, the welfare analysis proceeds in several steps. The first is to estimate the parameters of a utility function that describes how consumers will be affected by the changes in supply. We begin by assuming consumers are maximizing their utility based on the current supply and that, as harvests change, they will reoptimize. We specify a form for the utility function and use historical data to estimate its parameters. Projected changes in supply and real income are then plugged into the utility function to predict how consumers will respond. The estimated utility function tells us if consumers are better or worse off after the change and allows us to express the utility change in monetary terms.

Our model must be able to capture interactions between the demands for different species. If supply in one fishery falls over time, causing the price to increase, species with a stable or increasing supply become relatively cheaper and consumers are likely to substitute toward them. In this way, price effects and welfare impacts ripple through the system of demands. Modeling such interactions with a simultaneous system of demand equations becomes intractable as the number of species increases. To address the high dimensionality of the problem, we model demand in the 16 fisheries as a two-stage process in which consumers first allocate expenditures among the groups of related species, then further allocate expenditures among the species within those groups (Table 9).

Moore and Griffiths (2018) demonstrate how to estimate price changes and consumer welfare impacts in a multi-stage inverse demand system; we apply their approach to our projected changes in harvest. In a two-stage model, prices are determined by the consumers first allocating expenditures to fishery groups based on the aggregated supply in each, then among the individual species modeled in the second stage. A supply change in one fishery can affect the price of a species in a different group through the first-stage expenditure allocation. Consumer welfare impacts are found by measuring the distance between optimized consumption bundles in utility-space before and after the change in supply. The distance is then monetized using the forecasted expenditures on each fishery group. Real incomes are expected to grow through the end of the century; as their wealth increases, consumers will allocate some of that wealth to purchase the 16 modeled species, which will put an upward pressure on the prices. Our model captures this demand shift using an income elasticity for seafood from Cheng and Capps Jr.’s (1988) analysis of demand for seafood in the US and forecasts of changes in real income.

The scope of our analysis prevents us from collecting the data and developing the models required to forecast the change in harvest effort in response to stock changes and estimate producer surplus. While there are examples in the literature of studies that perform such analyses, they tend to focus on single fisheries. Edwards’ (2005) study of the Atlantic sea scallop fishery and Tan and Jardine’s (2019) analysis of the horseshoe crab fishery in the Delaware Bay are two examples. Performing that type of analysis on 16 different species for the entire US harvest is far beyond the scope of this paper. Markowski *et al.* (1999) limit their welfare analysis to consumer surplus when estimating the impact of climate change on the US commercial fishing industry for the same reason, as do Speers *et al.* (2016) in their analysis of coral reef-dependent fisheries under climate change and ocean acidification.

The assumption of an exogenous supply is not uncommon and has a long tradition in the agricultural and fisheries economics literatures (Moschini and Vissa, 1992; Eales and Unnevehr, 1994). Lacking production and cost functions to predict responses of harvesters, we make the simplifying assumption that harvest changes in proportion to thermally available habitat. This implies that in each fishery the fishery management authority imposes management measures, based on stock assessments, which aim to constrain the annual catch to a sustainable level, either directly through binding quotas or indirectly through limits on the fishing effort. The fishery stocks we examine here are managed by various regional councils, and the restrictions governing harvest are complex. Nonetheless, based on a review of the management measures currently in effect for all 16 species, we find strong empirical support for the constraints on catch or effort set according to biological criteria. The fishery management plans for 15 out of the 16 species we model are designed with the goal of maintaining either maximum sustainable yield, optimum sustainable yield, or some other biological benchmark. This empirical support for our assumption of constraints on catch that will adjust with stock assessments allows us to estimate the change in harvest level independent of the economic details of each fishery.

### 3.2. Estimation

The specific functional form we choose for the demand system is the inverse almost ideal demand system (Moschini and Vissa, 1992, see the estimating equations in Appendix). It is derived in a utility theoretic framework and estimated to satisfy adding-up and homogeneity restrictions. Seemingly unrelated regressions (SURs) are used to estimate the demand systems because there are no cross-equation restrictions on the estimated parameters but the error terms within a demand system are likely correlated. Data to estimate the model is the same as that which was used to perform the screening analysis, 2007–2016 NMFS Commercial Fishing Statistics; however, the original monthly observational units were maintained to capture seasonal variation in harvest and dockside price.

In the first-stage estimation of demand equations for the fishery groups, there are 12 estimated parameters; an additional 12 are identified by the utility theoretic restrictions and found using the estimated parameters. These parameters do not have a straightforward or intuitive interpretation; instead, we present the own-price elasticities for each fishery group (Table 12; the cross-price elasticities are shown in Appendix). An own-price elasticity tells us how much, in proportional terms, demand for a good is expected to change given a change in its price, assuming all other prices are held constant. Given the downward sloping demand curves, we expect these elasticities to be negative, providing a useful check on our model. The elasticities are nonlinear functions of the estimated utility parameters; given a sufficiently large sample to assume normality of the means, we employ the delta method to find the inner 95th percentiles as an indication of statistical significance. The first-stage price elasticities are all negative, of reasonable magnitude, and precisely estimated. All cross-price elasticities and the formula we use to find them are reported in Appendix.

Estimation of the second-stage demand systems proceeds exactly like the first stage. In the second stage, we estimate four separate demand systems via SURs using the total expenditures and harvest data for the constituent species of each fishery group (Table 13). As with the first stage, all own-price elasticity estimates are negative and, with the lone exception of Pacific halibut in the high-value fishery group, all 95% confidence intervals lie entirely below zero.

### 3.3. Forecast of total expenditures

Real income is expected to grow through the end of the century (Chen *et al.*, 2015). As consumers become wealthier, their demand, and thus willingness to pay, for normal goods increases. This has the effect of magnifying welfare impacts of changes in supply, whether positive or negative. To capture the effect of growing real income in our model, we take an estimated income elasticity of demand for seafood from the literature and forecast the change in total expenditures on the modeled fisheries until the end of our time horizon. Cheng and Capps Jr. (1988) estimate an income elasticity of demand for seafood of 0.11. Using the CIRA 2.0 gross domestic product (GDP) forecast produced by the MIT EPPA6 model (Chen *et al.*, 2015), we project real expenditures on the modeled species to grow by 16.5% by the end of the century (Table 14).

### 3.4. Forecast of price changes and consumer welfare impacts

Prices for each of the modeled species are forecasted by simulating the two-stage budget allocation process represented by our economic model. The first-stage demand system provides the forecasts of expenditure shares among the fishery groups, which are multiplied by the total expenditures in Table 14 to simulate budget allocation among those groups. The estimated second-stage demand systems then provide the means to further allocate group expenditures among the species which, when divided by forecasted harvests, provide the prices for each species. Table 15 shows the expected percentage changes in prices under the two modeled climate scenarios. Tables 10 and 11 show that projections for species harvest changes are mixed and one might expect the impact on prices to be similarly mixed given supply and price tend to be inversely related. The expectation that real expenditures will grow over time, however, puts upward pressure on all prices; as a result, prices are expected to increase under the RCP 4.5 scenario, and all but one price is expected to increase under the RCP 8.5 scenario. Additionally, because we explicitly model substitution, when an increase in the supply of one species makes it relatively cheaper, consumers will substitute toward that species, increasing the demand and dampening the downward pressure on price in economic equilibrium. For example, in the shrimp and shellfish group, white shrimp harvests are expected to increase by nearly 50% in the high emissions scenario, while their price is expected to stay about the same. This can be explained by observing that the share of shrimp and shellfish expenditures allocated to white shrimp grows from 27% to 37%, squeezing out some expenditures on scallops as their harvest falls by 20%. This one example shows how important demand interactions can be when modeling consumer welfare impacts.

We estimate the consumer welfare impacts using a distance function approach (Moore and Griffiths, 2018; Kim1997, see Appendix for derivation). The distance function is dual to the expenditure function and measures how the consumption bundle must be scaled to reach a reference level of utility. If we benchmark utility using the current harvest levels, we can find the change in consumer utility when the harvest levels change over time and monetize those changes using the expenditure forecasts. Forecasted prices are not used in the welfare calculation directly, but they embody much of the same information. Since our model explicitly accounts for substitution possibilities among the 16 modeled species, the net price elasticity reflects how changes in relative prices among the goods affect consumers’ willingness to pay for a given year’s harvest. Likewise, welfare impacts of a decline in harvest of one species can be mitigated if a substitute for that species experiences an increase in harvest. As such, there are some cases in which the change in harvest and the change in price have the same sign, despite all own-price elasticities being negative.

**Figure 9.**
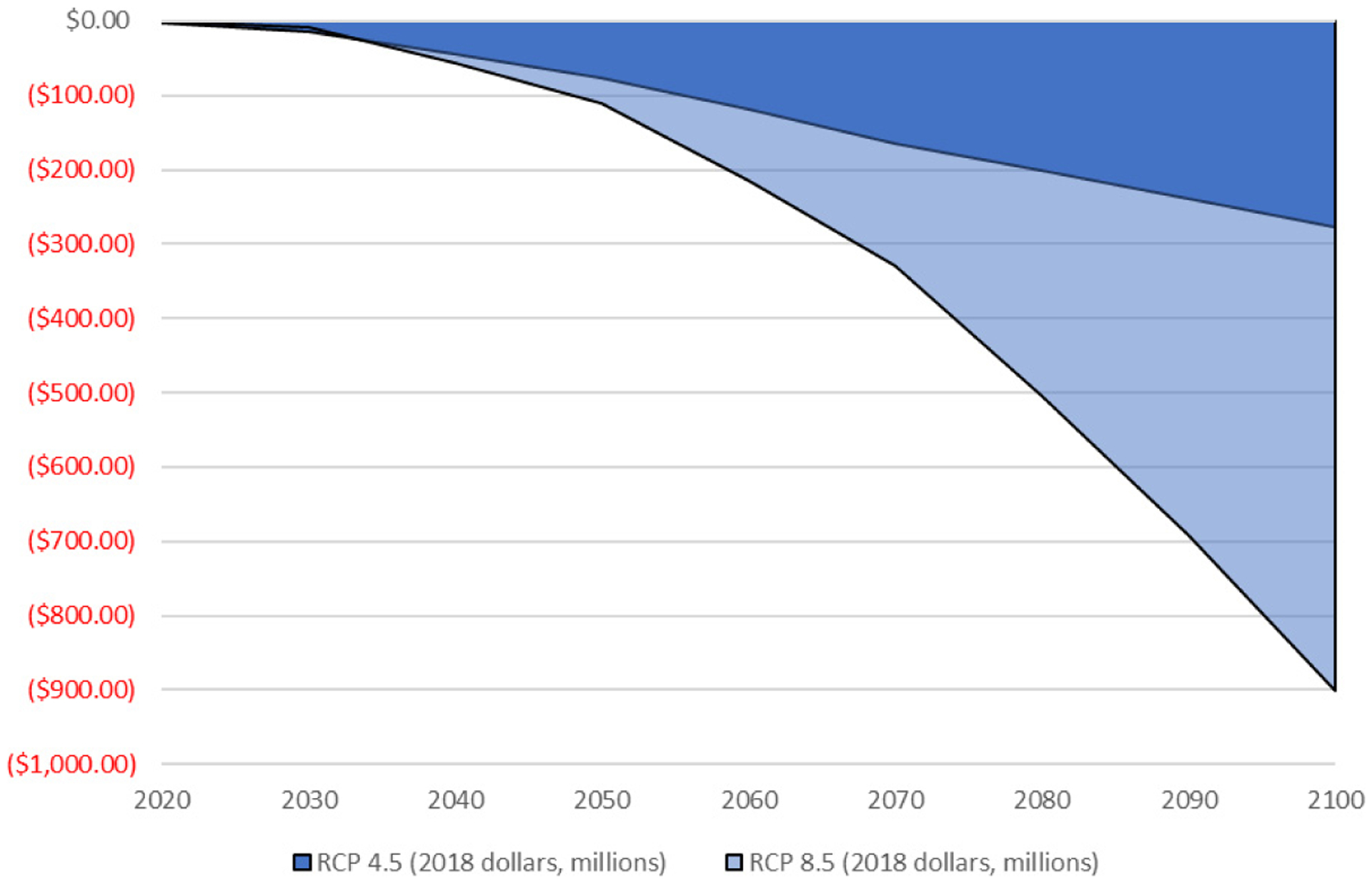
Annual consumer welfare impacts, RCP 4.5 versus RCP 8.5.

Table 16 shows the net present values of consumer welfare impacts through the end of the century for each of the fishery groups. Total welfare impacts are found by summing across groups and amount to a loss of $2.1 billion or $4.2 billion, depending on the climate scenario, when discounted at 3%. Table 17 and Fig. 9 show the total annual consumer welfare impacts at 10-year intervals through the end of the century. In the year 2050, the predicted annual consumer welfare losses reach $76 million in the low emissions scenario and $110 million in the high emissions scenario. By 2100, those losses reach $278 million in the lower emissions scenario and $901 million in the high emissions scenario.

## 4. Summary and Conclusions

Projections of changes in thermal habitat for marine species on the North American continental shelf predict major shifts in distribution by the end of the 21st century (Morley *et al.*, 2018). Changes in the extent of thermally available habitat are not necessarily predictive of the changes in the absolute biomass of any stock; a variety of other factors — such as the changes in ocean circulation patterns, ocean acidity, or nutrient loads — may affect population productivity. Nonetheless, changes in thermally available habitat for commercially harvested species are an important indicator of potential changes in abundance and, by extension, potential changes in the commercial harvest and landings.

The analysis presented here relies on the projected changes in thermally available habitat for marine species within the US EEZ to examine the implications of climate change for landings in 16 fisheries that together account for 56% of the US commercial fishing revenues. It suggests that for some species (e.g., blue crab, white shrimp, California market squid, and summer flounder) rising sea temperatures are likely to have a positive effect on landings, while for others (e.g., Dungeness crab, Florida stone crab, yellowfin sole, and chum salmon) the effect will be negative. The projected changes in landings under RCP 8.5 are generally more pronounced than those under RCP 4.5, particularly toward the end of the century; for some species, the projected increase or decrease in landings by 2100 exceeds 50%. Shifts of this magnitude suggest substantial changes by the end of the century in the distribution of species the US commercial fishing industry will harvest.

To estimate the consumer welfare impacts of projected changes in commercial landings, we employ a two-stage inverse demand model, building on the work previously conducted by Moore and Griffiths (2018). This approach captures interactions between the demands for different species, recognizing consumers’ ability to adapt to changes in market conditions and optimize their utility by reallocating their expenditures. The analysis suggests a positive impact on consumer welfare in the shrimp/mollusk sector but negative effects overall, particularly in the market for lobster and crab. The present value of the loss in consumer surplus from 2021 to 2100 is estimated at approximately $2.1 billion (2018 US$) under RCP 4.5 and $4.2 billion under RCP 8.5. The projection of annual losses grows with time and ranges from $278 million to $901 million by the end of the century.

This analysis extends the CIRA project’s body of work on the economic impacts of climate change and enhances our understanding of the effects of projected shifts in species distribution on the substantial segments of the commercial fishing industry. Additional research is needed, however, to develop a more comprehensive assessment. Areas of potential focus include consideration of additional species; expansion of the geographic scope of the analysis to include higher latitudes, particularly off the coast of Alaska; development of improved thermal niche models for species like white shrimp and blue crab, which at present may not effectively capture the species’ upper temperature limits; analysis of the potential impact of changes in ocean conditions (e.g., ocean acidification) on species productivity; and evaluation of the potential effects of changes in fisheries management (Kennedy, 2016; Gaines *et al.*, 2018). Other dynamics, such as growth in aquaculture or in international trade, may also have an important effect on the supply of seafood available to US consumers and are factors worth considering in subsequent assessments.

Beyond these considerations, it may be important to examine the cost to the commercial fishing industry of adapting to shifts in the distribution and abundance of target species. Changes of the magnitude projected in this analysis suggest that future generations of commercial fishermen may find themselves in waters very different from those fished by their predecessors. Adapting to these changes will challenge their knowledge and skills and may affect their capital and operating costs in ways that are difficult to predict, potentially affecting the vessels they operate, the gear they use, the fuel they consume, and the amount of time they spend at sea (Rogers *et al.*, 2019). It may also require additional investments in the infrastructure that supports the industry and processes its catch. This could prove to be particularly important if the shift in species distribution prompts demand for development or expansion of ports and processing facilities in more northern areas of Alaska. It also suggests that the importance of the industry to regional economies could change substantially over the next 80 years, and that the distribution of landings within and across regions could look quite different when the next century begins.

## Supplementary Material

Appendix

## Figures and Tables

**Table 1.** Commercial fishing landings by region, 2007–2016.

Region	Average annual weight (lb, billions)	Average annual value (2018 US$, billions)
East Coast	1.4	1.9
Gulf Coast	1.4	0.9
West Coast	1.1	0.7
Alaska	5.2	1.8
Total	9.2	5.3

**Table 2.** Matching of NMFS records to available habitat projections.

Status	Disposition	Count	Percentage of average annual ex-vessel value, 2007–2016
Match	Automated match	247	66%
	Manual match	5	4.8%
	Subtotal	252	70.8%
No match/Excluded	Multi-species records	88	7.8%
	No habitat projection	129	21.3%
	*De minimis* revenues	414	0.1%
	Subtotal	631	29.2%
Total		883	100%

**Table 3.** Coverage of high-value fisheries.

Rank	Fishery	Region(s)	Average annual ex-vessel revenues, 2007–2016 (2018 US$, millions)	Habitat projection available
1	American lobster	East Coast	502.5	Yes
2	Sea scallop	East Coast	501.4	Yes
3	Walleye pollock	West Coast and Alaska	390.4	Yes
4	Sockeye salmon	West Coast and Alaska	272.9	No
5	White shrimp	East Coast and Gulf Coast	246.3	Yes
6	Brown shrimp	East Coast and Gulf Coast	216.2	Yes
6	Pacific cod	West Coast and Alaska	212.1	Yes
7	Blue crab	East Coast and Gulf Coast	209.4	Yes
8	Dungeness crab	West Coast and Alaska	186.1	Yes
9	Pacific halibut	West Coast and Alaska	182.9	Yes
10	Sablefish	West Coast and Alaska	140	Yes

**Table 4.** Coverage of commercial fishing revenue by region.

	Average annual ex-vessel revenues, 2007–2016 (2018 US$, billions)		
Region	All fisheries	Fisheries with habitat projections	Percentage of area’s total revenue
East Coast	1.906	1.519	79.7%
Gulf Coast	0.885	0.567	64.1%
Subtotal: Atlantic	2.792	2.086	74.7%
West Coast	0.693	0.443	63.9%
Alaska	1.848	1.247	67.5%
Subtotal: Pacific	2.541	1.689	66.5%
Total	5.333	3.775	70.8%

**Table 5.** Results of screening analysis: RCP 4.5.

Region	Average annual ex-vessel revenues, 2007–2016 (2018 US$, billions)	Change in present value of ex-vessel revenues, 2021–2100: RCP 4.5 versus baseline, *r* = 3%	
		Projected change (2018 US$, billions)^a^	Percentage change
East Coast	1.519	0.415	0.9%
Gulf Coast	0.567	0.472	2.7%
Subtotal: Atlantic	2.086	0.887	1.4%
West Coast	0.443	(1.327)	−9.9%
Alaska	1.247	(0.598)	−1.6%
Subtotal: Pacific	1.689	(1.925)	−3.8%
Total	3.775	(1.037)	−0.9%

a For the purposes of screening analysis, the ex-vessel prices are held constant. The projected change in the present value of ex-vessel revenues assumes that the catch of each species analyzed would increase or decrease over time in direct proportion to the projected change in the species’ available habitat.

**Table 6.** Results of screening analysis: RCP 8.5.

Region	Average annual ex-vessel revenues, 2007–2016 (2018 US$, billions)	Change in present value of ex-vessel revenues, 2021–2100: RCP 8.5 versus baseline, *r* = 3%	
		Projected change (2018 US$, billions)^a^	Percentage change
East Coast	1.519	(0.116)	−0.3%
Gulf of Mexico	0.567	1.130	6.6%
Subtotal: Atlantic	2.086	1.014	1.6%
West Coast	0.443	(1.988)	−14.8%
Alaska	1.247	(0.649)	−1.7%
Subtotal: Pacific	1.689	(2.636)	−5.2%
Total	3.775	(1.623)	−1.4%

a For the purposes of screening analysis, the ex-vessel prices are held constant. The projected change in the present value of ex-vessel revenues assumes that the catch of each species analyzed would increase or decrease over time in direct proportion to the projected change in the species’ available habitat.

**Table 7.** Results of screening analysis for 20 highest-value fisheries: RCP 4.5.

Fishery	Annual ex-vessel revenues, 2007–2016 (2018 US$, millions)		Change in present value of ex-vessel revenues, 2021–2100: RCP 4.5 versus baseline, *r* = 3%	
	Mean	Standard deviation	Projected change (2018 US$, millions)^a^	Percentage change
American lobster	502.5	111.5	54.1	0.4%
Sea scallop	501.4	72.8	410.4	2.7%
Walleye pollock	390.4	49.9	79.9	0.7%
White shrimp	246.3	30.1	670.2	9%
Brown shrimp	216.2	51.6	(133.3)	−2%
Pacific cod	212.1	56.9	13	0.2%
Blue crab	209.4	19.6	407.8	6.4%
Dungeness crab	186.1	48	(800.4)	−14.2%
Pacific halibut	182.9	60.3	52	0.9%
Sablefish	140	27.3	227.6	5.4%
Snow crab	113	38.4	(1048.4)	−30.6%
Chum salmon	73.3	20.1	(210.9)	−9.5%
California market squid	58	22	132.3	7.5%
Chinook salmon	51.8	15.1	(36.3)	−2.3%
Yellowfin sole	49.2	10.5	(180.4)	−12.1%
Pacific hake	46.6	17.9	(51.6)	−3.7%
Ocean shrimp	34.2	20.6	(185)	−17.9%
Summer flounder	31.3	3.6	80.7	8.5%
Longfin squid	29.7	9.2	79.3	8.8%
Florida stone crab (claws)	28.7	5	(164.9)	−19%

a For the purposes of screening analysis, the ex-vessel prices are held constant. The projected change in the present value of ex-vessel revenues assumes that the catch of each species analyzed would increase or decrease over time in direct proportion to the projected change in the species’ available habitat.

**Table 8.** Results of screening analysis for 20 highest-value fisheries: RCP 8.5.

Fishery	Annual ex-vessel revenues, 2007–2016 (2018 US$, millions)		Change in present value of ex-vessel revenues, 2021–2100: RCP 8.5 versus baseline, *r* = 3%	
	Mean	Standard deviation	Projected change (2018 US$, millions)^a^	Percentage change
American lobster	502.5	111.5	(219.9)	−1.4%
Sea scallop	501.4	72.8	(114.3)	−0.8%
Walleye pollock	390.4	49.9	189.9	1.6%
White shrimp	246.3	30.1	1269.8	17%
Brown shrimp	216.2	51.6	(176.5)	−2.7%
Pacific cod	212.1	56.9	26.8	0.4%
Blue crab	209.4	19.6	649.2	10.2%
Dungeness crab	186.1	48	(1160.5)	−20.6%
Pacific halibut	182.9	60.3	28.9	0.5%
Sablefish	140	27.3	403.6	9.5%
Snow crab	113	38.4	(1549.8)	−45.3%
Chum salmon	73.3	20.1	(425.6)	−19.2%
California market squid	58	22	205.7	11.7%
Chinook salmon	51.8	15.1	24.3	1.5%
Yellowfin sole	49.2	10.5	(349)	−23.4%
Pacific hake	46.6	17.9	(78.5)	−5.6%
Ocean shrimp	34.2	20.6	(258.2)	−24.9%
Summer flounder	31.3	3.6	82.4	8.7%
Longfin squid	29.7	9.2	76	8.4%
Florida stone crab (claws)	28.7	5	(204.3)	−23.5%

a For the purposes of screening analysis, the ex-vessel prices are held constant. The projected change in the present value of ex-vessel revenues assumes that the catch of each species analyzed would increase or decrease over time in direct proportion to the projected change in the species’ available habitat.

**Table 9.** Species selected for welfare analysis.

Fishery	Annual ex-vessel revenues, 2007–2016 (2018 US$, millions)		Baseline rank by value	Fishery group
	Mean	Standard deviation		
American lobster	502.5	111.5	1	Lobster/crab
Sea scallop	501.4	72.8	2	Shrimp/mollusk
Walleye pollock	390.4	49.9	3	Low-value fish
White shrimp	246.3	30.1	4	Shrimp/mollusk
Brown shrimp	216.2	51.6	5	Shrimp/mollusk
Pacific cod	212.1	56.9	6	Low-value fish
Blue crab	209.4	19.6	7	Lobster/crab
Dungeness crab	186.1	48	8	Lobster/crab
Pacific halibut	182.9	60.3	9	High-value fish
Sablefish	140	27.3	10	High-value fish
Chum salmon	73.3	20.1	12	Low-value fish
California market squid	58	22	13	Shrimp/mollusk
Chinook salmon	51.8	15.1	14	High-value fish
Yellowfin sole	49.2	10.5	15	Low-value fish
Summer flounder	31.3	3.6	18	High-value fish
Florida stone crab (claws)	28.7	5	20	Lobster/crab
Total	3079.6			

**Table 10.** Projected changes in commercial harvests for the 16 modeled species, RCP 4.5.

Fishery group	Fishery	Annual average, 2007–2016 (MM lb)	2050 Projection^a^			2090 Projection^a^		
			Landings (MM lb)	Percentage change	95% Confidence interval	Landings (MM lb)	Percentage change	95% Confidence interval
Lobster/crab	Blue crab	168.3	180.1	7.1%	2.4–12.7%	194.5	15.6%	4.4–28.1%
	Dungeness crab	58.9	49.2	(16.5%)	(25.2–7.1%)	41	(30.4%)	(41.9–18.6%)
	American lobster	126.7	128.3	1.3%	(3.1)-5.1%	121.1	(4.4%)	(11.3)-2.1%
	Florida stone Crab (claws)	4.6	3.5	(25.1%)	(34.6–13.3%)	3	(35.6%)	(48.7–20.1%)
Shrimp/mollusk	Sea scallop	49.3	51.5	4.3%	0.4–9.2%	49.2	(0.3%)	(4.9)-3.9%
	Brown shrimp	107.4	104.8	(2.5%)	(5.6)-0.2%	102.7	(4.4%)	(9.6)-0.4%
	White shrimp	109.1	120.8	10.7%	5.8–15.2%	127.7	17%	9–24.1%
	California market squid	179.4	193.9	8%	6.1–10.4%	215	19.8%	15.6–26.1%
High-value fish	Pacific halibut	43.2	43.1	(0.2%)	(1.8)-1.7%	44.8	3.6%	1.9–5.8%
	Sablefish	39.6	42.1	6.2%	(1.9)-15%	44.6	12.5%	5.7–21.5%
	Chinook salmon	14.5	13.9	(3.7%)	(8.6)-1.3%	14.3	(0.8%)	(6.5)-5.1%
	Summer flounder	11.2	12.4	10.2%	2.1–19.4%	13	15.6%	3.3–29.4%
Low-value fish	Walleye pollock	2760.6	2765.1	0.2%	(1.4)-1.1%	2840.3	2.9%	1.1–4.5%
	Pacific cod	620.2	616.4	(0.6%)	(1.8)-0.6%	631.2	1.8%	(1.6)-5.1%
	Yellowfin sole	290.6	257.7	(11.3%)	(21.9)-0%	200.4	(31%)	(44.4–19%)
	Chum salmon	118.7	106.8	(10.1%)	(18)-0.6%	84.5	(28.8%)	(38–20.1%)

a The projected change in commercial harvests assumes that the catch of each species would increase or decrease in direct proportion to the projected change in the species’ thermally available habitat.

**Table 11.** Projected changes in commercial harvests for the 16 modeled species, RCP 8.5.

Fishery group	Fishery	Annual average, 2007–2016 (MM lb)	2050 Projection^a^			2090 Projection^a^		
			Landings (MM lb)	Percentage change	95% Confidence interval	Landings (MM lb)	Percentage change	95% Confidence interval
Lobster/crab	Blue crab	168.3	179.1	6.4%	3.1–9.7%	250.5	48.9%	28.3–69.9%
	Dungeness crab	58.9	45.6	(22.5%)	(35.2–11.3%)	23	(60.9%)	(74.4–44.9%)
	American lobster	126.7	129.2	1.9%	(3.2)-6.1%	101.5	(19.9%)	(30.5–12.2%)
	Florida stone crab (claws)	4.6	3.3	(29.3%)	(39.7–16.2%)	2.1	(54%)	(68.9–34.7%)
Shrimp/mollusk	Sea scallop	49.3	51.2	3.8%	(6.1)-12.8%	39.5	(20%)	(32.5–6.9%)
	Brown shrimp	107.4	103.4	(3.7%)	(7.7)-0.3%	101.9	(5.2%)	(12.7)-2.4%
	White shrimp	109.1	127.8	17.1%	13.2–20.6%	161.8	48.2%	35.6–60.7%
	California market squid	179.4	201.6	12.3%	10.1–15.3%	241.4	34.6%	27.2–46.2%
High-value fish	Pacific halibut	43.2	44.1	2.1%	(1.3)-5.2%	39.4	(8.7%)	(23.1)-3%
	Sablefish	39.6	45.1	13.8%	7.9–20.1%	42.8	8.1%	(10.4)-23.7%
	Chinook salmon	14.5	14.8	2.5%	(6.7)-13.8%	15.4	6.2%	(14.6)-35%
	Summer flounder	11.2	12.3	9.2%	2.2–16.9%	14.5	28.8%	12.2–44.2%
Low-value fish	Walleye pollock	2760.6	2840.4	2.9%	1–4.7%	2733.3	(1%)	(10.1)-7.9%
	Pacific cod	620.2	633.9	2.2%	(0.4)-4.6%	582.8	(6%)	(18.5)-6.5%
	Yellowfin sole	290.6	213.3	(26.6%)	(37.2–18%)	92.5	(68.2%)	(84.4–52.7%)
	Chum salmon	118.7	95.1	(19.8%)	(30.7–9.9%)	41.9	(64.7%)	(68.3–61.1%)

**Table 12.** First-stage own-price elasticities.

Fishery group	Price elasticity	Inner 95th percentile	
Lobster/crab	−0.554	−0.615	−0.493
Shrimp/mollusk	−0.754	−0.793	−0.714
High-value fish	−0.302	−0.344	−0.259
Low-value fish	−0.921	−0.939	−0.902

**Table 13.** Second-stage price elasticities.

Fishery group	Species	Price elasticity	Inner 95th percentile	
Lobster/crab	Blue crab	−0.290	−0.357	−0.224
	Dungeness crab	−0.263	−0.324	−0.203
	American lobster	−0.630	−0.664	−0.596
	Florida stone crab (claws)	−0.717	−0.733	−0.701
Shrimp/mollusk	Sea scallop	−0.461	−0.532	−0.390
	Brown shrimp	−0.418	−0.495	−0.342
	White shrimp	−0.418	−0.476	−0.361
	California market squid	−0.995	−1.008	−0.982
High-value fish	Pacific halibut	−0.081	−0.217	0.056
	Sablefish	−0.363	−0.457	−0.268
	Chinook salmon	−0.290	−0.356	−0.222
	Summer flounder	−0.878	−0.886	−0.868
Low-value fish	Walleye pollock	−0.590	−0.646	−0.533
	Pacific cod	−0.699	−0.725	−0.672
	Yellowfin sole	−0.335	−0.489	−0.182
	Chum salmon	−0.760	−0.791	−0.730

**Table 14.** Forecast of total expenditures on the modeled species.

Year	GDP (2018 US$, billions)	Expenditures on modeled species (2018 US$, millions)
2007–2016 average	16,674	3080
2020	19,090	3088
2030	23,859	3165
2040	29,253	3237
2050	35,051	3302
2060	41,589	3364
2070	48,864	3424
2080	56,849	3482
2090	65,477	3536
2100	74,688	3588

**Table 15.** Projected changes in prices.

Fishery group	Species	Baseline price (2018 US$)^a^	Percent change in price by 2100	
			RCP 4.5	RCP 8.5
Lobster/crab	Blue crab	1.17	33.35%	35.57%
	Dungeness crab	3.32	31.65%	21.47%
	Lobster	5.49	31.42%	62.07%
	Florida stone crab (claws)	5.59	55.83%	69.18%
Shrimp/mollusk	Sea scallop	9.35	19.4%	14.08%
	Brown shrimp	2.95	22.5%	21.46%
	White shrimp	2.87	14.72%	0.59%
	Market squid	0.30	5.75%	−5.30%
High-value fish	Pacific halibut	2.96	26.61%	18.99%
	Sablefish	2.79	16.64%	2.24%
	Chinook salmon	3.18	25.01%	5.98%
	Summer flounder	2.93	17.21%	5.09%
Low-value fish	Walleye pollock	0.14	5.06%	56.72%
	Pacific cod	0.32	4.69%	58.32%
	Yellowfin sole	0.08	32.21%	204.18%
	Chum salmon	0.51	19.35%	57.38%

a Baseline prices represent the average monthly price (2018 US$) for each species from 1996 to 2016.

**Table 16.** Present values of consumer welfare impacts (*r* = 3%).

Fishery group	RCP 4.5 (2018 US$, millions)	RCP 8.5 (2018 US$, millions)
Lobster/crab	−2126	−2848.2
Shrimp/mollusk	265.7	1469.7
High-value fish	−585.1	441.2
Low-value fish	355.8	−3292
Total	−2089.7	−4229.2

**Table 17.** Annual consumer welfare impacts, RCP 4.5 versus RCP 8.5.

Year	RCP 4.5 (2018 US$, millions)	RCP 8.5 (2018 US$, millions)
2020	−1.18	−0.55
2030	−14.21	−7.29
2040	−44.21	−56.45
2050	−75.82	−109.59
2060	−118.90	−215.36
2070	−164.22	−329.27
2080	−200.60	−504.22
2090	−238.51	−693.10
2100	−277.94	−901.25
