# Supplementary material for: ESTIMATING THE ECONOMIC IMPACTS OF CLIMATE CHANGE ON 16 MAJOR US FISHERIES
Source: Clim Chang Econ (Singap). Author manuscript; Available in PMC 2021 Feb 23. (PMC7900876; doi:10.1142/s2010007821500020)
Supplement: Appendix [file NIHMS1666338-supplement-Appendix.docx]

Appendix

**Estimating Equations**

The inverse almost ideal demand system (Moschini and Vissa 1992) uses the following estimating equation:

$$W_{i}=\alpha_{i}+ \text{ }\sum_{j}\gamma_{ij}ln\left(X_{j}\right)-\beta_{i}\ln Q$$

$W_{i}$= expenditure share for good *i*

$X_{j}$= quantity of good *j*

$\ln Q$=$\text{ }\sum_{i}\alpha_{i}ln\left(X_{i}\right)\text{ }+\text{ }\left(½\right)\sum_{i}\sum_{j}\gamma_{ij}ln\left(X_{i}\right)ln\left(X_{j}\right)$

$\alpha_{i}$, $\gamma_{ij}$, $\beta_{i}$ are estimated parameters

The price elasticity for good *i* with respect to the quantity of good *j* is:

$$E_{ij}=\frac{\partial\ln[P_{i}]}{\partial\ln[X_{j}]}=-\delta_{ij}+\left\{\gamma_{ij}-\beta_{i}\left(\bar{W}_{j}+ \beta_{j}\ln\bar{Q}\right)\right\}\left(\frac{1}{\bar{W}_{j}}\right)$$

$P_{i}$ = price of good *i*

$\delta_{ij}$ = kroneker delta, equals 1 if *i = j* and 0 otherwise

$\bar{W}_{j}$= sample mean of expenditure shares for good *j*

$\ln\bar{Q}$ = the average of the lnQ index, evaluated for the sample, as defined above

We use compensating surplus to estimate consumer welfare impacts. The formula for compensating surplus in an inverse almost ideal demand system is:

$$CS=\exp\left[\sum_{i}\alpha_{i}ln\left(X'_{i}\right)\text{ }+\text{ }\left(½\right)\sum_{i}\sum_{j}\gamma_{ij}ln\left(X'_{i}\right)ln\left(X'_{j}\right)-\frac{\sum_{i}\alpha_{i}ln\left(X_{i}\right)\text{ }+\text{ }\left(½\right)\sum_{i}\sum_{j}\gamma_{ij}ln\left(X_{i}\right)ln\left(X_{j}\right)}{\prod_{i} X_{i}^{\beta_{i}}}\prod_{i} X'_{i}^{\beta_{i}}\right]-1$$

where $X'$indicates the quantity of goods after the change.

**Estimation results**

The singularity of the cross-equation covariance matrix requires dropping one of the equations from the system and relying on the adding up, homogeneity and symmetry conditions to identify the parameters. So that,

$$\sum_{i} \alpha_{i}=1$$

$$\sum_{i} \beta_{i}=0$$

3. $\sum_{i} \gamma_{ij}=0$ and $\gamma_{ij}=\gamma_{ji}$

The stability of the parameters using this approach was confirmed by reestimating each of the inverse demand systems, omitting a different equation, and reproducing the same results. Homogeneity and symmetry restrictions are imposed by estimating a subset of the model parameters and solving for the remaining restricted parameters. The estimated parameters are found via seemingly unrelated regression (SUR).

**Table A1: First Stage**

Equation 1: Lobster and Crab

Equation 2: Shrimp and Shellfish

Equation 3: High Value Fish

Equation 4: Low Value Fish (omitted)

| Parameter | Estimate | Std. Error | z value | p value | 95% Conf. Interval | |
| --- | --- | --- | --- | --- | --- | --- |
| α_1_ | 0.1429 | 0.1377 | 1.040 | 0.300 | -0.1271 | 0.4128 |
| α_2_ | 0.1431 | 0.1606 | 0.890 | 0.373 | -0.1718 | 0.4579 |
| α_3_ | 0.2651 | 0.1004 | 2.640 | 0.008 | 0.0683 | 0.4620 |
| β_1_ | -0.0071 | 0.0080 | -0.880 | 0.380 | -0.0228 | 0.0087 |
| β_2_ | -0.0157 | 0.0094 | -1.670 | 0.094 | -0.0340 | 0.0027 |
| β_3_ | -0.0028 | 0.0059 | -0.480 | 0.633 | -0.0143 | 0.0087 |
| ϒ_11_ | 0.1488 | 0.0064 | 23.130 | 0.000 | 0.1362 | 0.1614 |
| ϒ_12_ | -0.0308 | 0.0052 | -5.930 | 0.000 | -0.0409 | -0.0206 |
| ϒ_13_ | -0.0856 | 0.0030 | -28.090 | 0.000 | -0.0916 | -0.0796 |
| ϒ_22_ | 0.0729 | 0.0067 | 10.800 | 0.000 | 0.0596 | 0.0861 |
| ϒ_23_ | 0.0075 | 0.0031 | 2.440 | 0.015 | 0.0015 | 0.0135 |
| ϒ_33_ | 0.1084 | 0.0029 | 37.570 | 0.000 | 0.1028 | 0.1141 |

**Table A2: Second Stage, Lobster and Crab**

Equation 1: Blue Crab

Equation 2: Dungeness Crab

Equation 3: Lobster

Equation 4: Stone Crab (omitted)

| Parameter | Estimate | Std. Error | z value | p value | 95% Conf. Interval | |
| --- | --- | --- | --- | --- | --- | --- |
| α_1_ | 0.7347 | 0.1111 | 6.610 | 0.000 | 0.5169 | 0.9525 |
| α_2_ | -0.8150 | 0.0772 | -10.560 | 0.000 | -0.9663 | -0.6638 |
| α_3_ | 1.0903 | 0.1191 | 9.160 | 0.000 | 0.8569 | 1.3237 |
| β_1_ | 0.0450 | 0.0072 | 6.250 | 0.000 | 0.0309 | 0.0591 |
| β_2_ | -0.0769 | 0.0049 | -15.550 | 0.000 | -0.0866 | -0.0672 |
| β_3_ | 0.0404 | 0.0076 | 5.290 | 0.000 | 0.0254 | 0.0554 |
| ϒ_11_ | 0.1858 | 0.0087 | 21.250 | 0.000 | 0.1687 | 0.2029 |
| ϒ_12_ | -0.1042 | 0.0101 | -10.280 | 0.000 | -0.1240 | -0.0843 |
| ϒ_13_ | -0.0605 | 0.0072 | -8.440 | 0.000 | -0.0745 | -0.0464 |
| ϒ_22_ | 0.2377 | 0.0127 | 18.690 | 0.000 | 0.2128 | 0.2626 |
| ϒ_23_ | -0.1504 | 0.0120 | -12.560 | 0.000 | -0.1739 | -0.1270 |
| ϒ_33_ | 0.2227 | 0.0156 | 14.240 | 0.000 | 0.1920 | 0.2533 |

**Table A3: Second Stage, Shrimp and Shellfish**

Equation 1: Sea Scallop

Equation 2: Brown Shrimp

Equation 3: White Shrimp

Equation 4: Market Squid (omitted)

| Parameter | Estimate | Std. Error | z value | p value | 95% Conf. Interval | |
| --- | --- | --- | --- | --- | --- | --- |
| α_1_ | 0.0514 | 0.1450 | 0.350 | 0.723 | -0.2327 | 0.3356 |
| α_2_ | 0.7807 | 0.1280 | 6.100 | 0.000 | 0.5298 | 1.0316 |
| α_3_ | -0.1591 | 0.1095 | -1.450 | 0.146 | -0.3736 | 0.0555 |
| β_1_ | -0.0321 | 0.0094 | -3.410 | 0.001 | -0.0506 | -0.0137 |
| β_2_ | 0.0373 | 0.0083 | 4.490 | 0.000 | 0.0210 | 0.0536 |
| β_3_ | -0.0230 | 0.0071 | -3.250 | 0.001 | -0.0369 | -0.0091 |
| ϒ_11_ | 0.2139 | 0.0121 | 17.640 | 0.000 | 0.1901 | 0.2376 |
| ϒ_12_ | -0.1066 | 0.0112 | -9.510 | 0.000 | -0.1286 | -0.0846 |
| ϒ_13_ | -0.0815 | 0.0052 | -15.670 | 0.000 | -0.0916 | -0.0713 |
| ϒ_22_ | 0.1589 | 0.0124 | 12.770 | 0.000 | 0.1346 | 0.1833 |
| ϒ_23_ | -0.0603 | 0.0069 | -8.770 | 0.000 | -0.0738 | -0.0469 |
| ϒ_33_ | 0.1532 | 0.0066 | 23.180 | 0.000 | 0.1402 | 0.1661 |

**Table A4: Second Stage, High Value Fish**

Equation 1: Pacific Halibut

Equation 2: Sablefish

Equation 3: Chinook Salmon

Equation 4: Summer Flounder (omitted)

| Parameter | Estimate | Std. Error | z value | p value | 95% Conf. Interval | |
| --- | --- | --- | --- | --- | --- | --- |
| α_1_ | -1.3784 | 0.1028 | -13.410 | 0.000 | -1.5798 | -1.1770 |
| α_2_ | 1.0824 | 0.1199 | 9.030 | 0.000 | 0.8475 | 1.3174 |
| α_3_ | 0.2310 | 0.0916 | 2.520 | 0.012 | 0.0515 | 0.4105 |
| β_1_ | -0.1155 | 0.0070 | -16.570 | 0.000 | -0.1292 | -0.1018 |
| β_2_ | 0.0638 | 0.0083 | 7.650 | 0.000 | 0.0475 | 0.0802 |
| β_3_ | 0.0041 | 0.0063 | 0.650 | 0.514 | -0.0082 | 0.0163 |
| ϒ_11_ | 0.3046 | 0.0243 | 12.510 | 0.000 | 0.2569 | 0.3523 |
| ϒ_12_ | -0.1528 | 0.0182 | -8.400 | 0.000 | -0.1884 | -0.1171 |
| ϒ_13_ | -0.0276 | 0.0105 | -2.630 | 0.008 | -0.0482 | -0.0071 |
| ϒ_22_ | 0.2202 | 0.0181 | 12.180 | 0.000 | 0.1848 | 0.2557 |
| ϒ_23_ | -0.0241 | 0.0066 | -3.670 | 0.000 | -0.0369 | -0.0112 |
| ϒ_33_ | 0.0636 | 0.0034 | 18.890 | 0.000 | 0.0570 | 0.0702 |

**Table A5: Second Stage, Low Value Fish**

Equation 1: Walleye Pollock

Equation 2: Pacific Cod

Equation 3: Chum Salmon

Equation 4: Yellowfin Sole (omitted)

| Parameter | Estimate | Std. Error | z value | p value | 95% Conf. Interval | |
| --- | --- | --- | --- | --- | --- | --- |
| α_1_ | 0.9638 | 0.0805 | 11.970 | 0.000 | 0.8060 | 1.1216 |
| α_2_ | 0.7753 | 0.0778 | 9.970 | 0.000 | 0.6228 | 0.9277 |
| α_3_ | 0.1944 | 0.0680 | 2.860 | 0.004 | 0.0612 | 0.3276 |
| β_1_ | 0.0464 | 0.0052 | 8.920 | 0.000 | 0.0362 | 0.0566 |
| β_2_ | 0.0247 | 0.0048 | 5.090 | 0.000 | 0.0152 | 0.0342 |
| β_3_ | 0.0051 | 0.0042 | 1.200 | 0.230 | -0.0032 | 0.0133 |
| ϒ_11_ | 0.2035 | 0.0153 | 13.260 | 0.000 | 0.1734 | 0.2335 |
| ϒ_12_ | -0.0620 | 0.0075 | -8.220 | 0.000 | -0.0768 | -0.0472 |
| ϒ_13_ | -0.0064 | 0.0063 | -1.020 | 0.310 | -0.0189 | 0.0060 |
| ϒ_22_ | 0.1279 | 0.0066 | 19.450 | 0.000 | 0.1150 | 0.1407 |
| ϒ_23_ | -0.0055 | 0.0037 | -1.500 | 0.134 | -0.0127 | 0.0017 |
| ϒ_33_ | 0.0237 | 0.0028 | 8.570 | 0.000 | 0.0183 | 0.0291 |

**Price Elasticities (Flexibilities)**

**Table A6: First Stage**

|  | Lobster Crab | Shrimp & Shellfish | High Value Fish | Low Value Fish |
| --- | --- | --- | --- | --- |
| Lobster Crab | -0.5061 | -0.0889 | -0.5980 | -0.1305 |
| Standard Error | 0.0218 | 0.0149 | 0.0217 | 0.0132 |
|  |  |  |  |  |
| Shrimp & Shellfish | -0.0921 | -0.7839 | 0.0630 | -0.1838 |
| Standard Error | 0.0194 | 0.0163 | 0.0222 | 0.0150 |
|  |  |  |  |  |
| High Value Fish | -0.2800 | 0.0225 | -0.2347 | -0.1340 |
| Standard Error | 0.0115 | 0.0093 | 0.0208 | 0.0098 |
|  |  |  |  |  |
| Low Value Fish | -0.1218 | -0.0996 | -0.2452 | -0.9162 |
| Standard Error | 0.0157 | 0.0193 | 0.0449 | 0.0056 |

**Table A7: Second Stage, Lobster and Crab**

|  | Blue Crab | Dungeness Crab | Lobster | Stone Crab |
| --- | --- | --- | --- | --- |
| Blue Crab | -0.3552 | -0.4083 | -0.1980 | -0.4107 |
| Standard Error | 0.0206 | 0.0365 | 0.0105 | 0.0762 |
|  |  |  |  |  |
| Dungeness Crab | -0.2591 | -0.0473 | -0.2124 | 0.3325 |
| Standard Error | 0.0269 | 0.0420 | 0.0161 | 0.0937 |
|  |  |  |  |  |
| Lobster | -0.3189 | -0.6098 | -0.5767 | -0.2298 |
| Standard Error | 0.0299 | 0.0430 | 0.0201 | 0.0797 |
|  |  |  |  |  |
| Stone Crab | -0.0667 | -0.0974 | -0.0333 | -0.9769 |
| Standard Error | 0.0114 | 0.0241 | 0.0064 | 0.0056 |

**Table A8: Second Stage, Shrimp and Shellfish**

|  | Sea Scallop | Brown Shrimp | White Shrimp | Market Squid |
| --- | --- | --- | --- | --- |
| Sea Scallop | -0.4595 | -0.3852 | -0.2670 | -0.4560 |
| Standard Error | 0.0370 | 0.0352 | 0.0230 | 0.0746 |
|  |  |  |  |  |
| Brown Shrimp | -0.2901 | -0.4146 | -0.2569 | 0.1109 |
| Standard Error | 0.0336 | 0.0404 | 0.0295 | 0.0726 |
|  |  |  |  |  |
| White Shrimp | -0.1717 | -0.2129 | -0.4173 | -0.1906 |
| Standard Error | 0.0168 | 0.0220 | 0.0297 | 0.0621 |
|  |  |  |  |  |
| Market Squid | -0.0787 | -0.1203 | -0.1117 | -0.9947 |
| Standard Error | 0.0140 | 0.0208 | 0.0193 | 0.0065 |

**Table A9: Second Stage, High Value Fish**

|  | Pacific Halibut | Sablefish | Chinook Salmon | Summer Flounder |
| --- | --- | --- | --- | --- |
| Pacific Halibut | -0.0605 | -0.3690 | -0.1927 | -0.3907 |
| Standard Error | 0.0719 | 0.0530 | 0.1112 | 0.0681 |
|  |  |  |  |  |
| Sablefish | -0.4762 | -0.3558 | -0.3359 | -0.2500 |
| Standard Error | 0.0560 | 0.0499 | 0.0702 | 0.0356 |
|  |  |  |  |  |
| Chinook Salmon | -0.0797 | -0.0825 | -0.2895 | -0.0538 |
| Standard Error | 0.0341 | 0.0159 | 0.0343 | 0.0083 |
|  |  |  |  |  |
| Summer Flounder | -0.3836 | -0.4549 | -1.4455 | -0.8775 |
| Standard Error | 0.0563 | 0.0668 | 0.2057 | 0.0047 |

**Table A10: Second Stage, Low Value Fish**

|  | Walleye Pollock | Pacific Cod | Yellowfin Sole | Chum Salmon |
| --- | --- | --- | --- | --- |
| Walleye Pollock | -0.6094 | -0.2200 | -0.2618 | -0.9258 |
| Standard Error | 0.0249 | 0.0185 | 0.1566 | 0.0942 |
|  |  |  |  |  |
| Pacific Cod | -0.1782 | -0.7047 | -0.1976 | -0.4066 |
| Standard Error | 0.0118 | 0.0127 | 0.0979 | 0.0703 |
|  |  |  |  |  |
| Yellowfin Sole | -0.0223 | -0.0207 | -0.3381 | -0.0787 |
| Standard Error | 0.0090 | 0.0077 | 0.0789 | 0.0539 |
|  |  |  |  |  |
| Chum Salmon | -0.1901 | -0.2460 | -3.6928 | -0.7602 |
| Standard Error | 0.0250 | 0.0356 | 0.4111 | 0.0155 |
